# Predicting Optimal Antimalarial Drug Combinations from a Standardized Plasmodium falciparum Humanized Mouse Model

**DOI:** 10.1128/aac.01574-22

**Published:** 2023-05-03

**Authors:** Claudia Demarta-Gatsi, Nicole Andenmatten, María-Belén Jiménez-Díaz, Nathalie Gobeau, Mohammed H. Cherkaoui-Rabti, Aline Fuchs, Pablo Díaz, Sandra Berja, Rebeca Sánchez, Hazel Gómez, Estíbaliz Ruiz, Paula Sainz, Eider Salazar, Rodrigo Gil-Merino, Luis Manuel Mendoza, Cristina Eguizabal, Didier Leroy, Joerg J. Moehrle, Belen Tornesi, Iñigo Angulo-Barturen

**Affiliations:** a Medicines for Malaria Venture, Geneva, Switzerland; b The Art of Discovery, Derio, Basque Country, Spain; c Cell Therapy, Stem Cells and Tissues Group, Biocruces Bizkaia Health Research Institute, Barakaldo, Bizkaia, Spain; d Basque Centre for Blood Transfusion and Human Tissues, Galdakao, Bizkaia, Spain

**Keywords:** *Plasmodium falciparum*, antimalarial agents, drug combinations, drug discovery, humanized mouse, *in vivo* pharmacology, preclinical drug studies

## Abstract

The development of new combinations of antimalarial drugs is urgently needed to prevent the spread of parasites resistant to drugs in clinical use and contribute to the control and eradication of malaria. In this work, we evaluated a standardized humanized mouse model of erythrocyte asexual stages of Plasmodium falciparum (PfalcHuMouse) for the selection of optimal drug combinations. First, we showed that the replication of P. falciparum was robust and highly reproducible in the PfalcHuMouse model by retrospective analysis of historical data. Second, we compared the relative value of parasite clearance from blood, parasite regrowth after suboptimal treatment (recrudescence), and cure as variables of therapeutic response to measure the contributions of partner drugs to combinations *in vivo*. To address the comparison, we first formalized and validated the day of recrudescence (DoR) as a new variable and found that there was a log-linear relationship with the number of viable parasites per mouse. Then, using historical data on monotherapy and two small cohorts of PfalcHuMice evaluated with ferroquine plus artefenomel or piperaquine plus artefenomel, we found that only measurements of parasite killing (i.e., cure of mice) as a function of drug exposure in blood allowed direct estimation of the individual drug contribution to efficacy by using multivariate statistical modeling and intuitive graphic displays. Overall, the analysis of parasite killing in the PfalcHuMouse model is a unique and robust experimental *in vivo* tool to inform the selection of optimal combinations by pharmacometric pharmacokinetic and pharmacodynamic (PK/PD) modeling.

## INTRODUCTION

Malaria is a global infectious disease caused by parasitic protozoa of the genus *Plasmodium* that infect, feed on, and destroy human erythrocytes (1). Malaria is transmitted by *Anopheles* mosquitoes; the global malaria burden in 2020 was about 241 million new cases causing 627,000 deaths, 90% of which occurred in sub-Saharan African children under 5 years and pregnant women (2). Despite the fact that malaria is a curable disease, resistance to classical antimalarials in clinical use (e.g., amodiaquine, chloroquine, mefloquine, quinine, and sulfadoxine-pyrimethamine) emerged and spread during the second half of 20th century, when these drugs were used as monotherapies (3). To counter the spread of resistance, in 2001 the World Health Organization (WHO) recommended the use of fixed-dose combinations of artemisinin with a second antimalarial (artemisinin combination therapies [ACTs]) only for the treatment of uncomplicated cases. In spite of this recommendation, resistance against artemisinins emerged and spread in 2007 in Southeast Asia and recently in East Africa (4), lending great urgency to discover new classes of antimalarials (5).

The development and rational use of effective new drug combinations are important goals of global health initiatives to avoid the loss of effective drugs for clinical use (6). However, developing drugs as combinations is challenging from the pharmaceutical development, regulatory, and experimental points of view. These challenges include, for instance, problems associated with excessive pill size, coformulability, drug-drug interaction risks, development of combinations with drugs that have never been deployed as monotherapies in the clinic and the large number of plausible drugs to be combined, and potential schedules of dosing to be explored. Therefore, developing and improving new and existing experimental and modeling tools to mitigate the aforementioned risks are urgent necessities.

The selection of partner drugs in combinations has been classically addressed by using *in vitro* cell culture systems, which allowed testing large numbers of candidate combinations in different ratios (7–9). However, these systems cannot reproduce the complex physiological and pharmacokinetic (PK)-pharmacodynamic (PD) interactions that take place *in vivo* among drugs, hosts, and pathogens that are critical for understanding the antimalarial efficacy and translatability of drug testing endpoints among different species. Even though preclinical *in vivo* studies have decisively contributed to the identification of effective combinations of standard and new drugs for most infectious diseases, including malaria (10, 11), practical limitations have resulted in an underexploitation of *in vivo* models to explore the pharmacological properties of new antimalarial combinations. In general, these limitations were as follows: first, the ethical concerns associated with the large number of individuals necessary to conduct statistically sound studies and the use of nonhuman primates, particularly, endangered species; second, the logistical difficulties of *in vivo* studies (e.g., large amounts of drug necessary, practical difficulties to obtain homogeneous groups of animals); and third, the lack of standardized methods of data analysis to gauge and compare the contributions of partner drugs in combinations, particularly when the parasites used were not human pathogens (e.g., Plasmodium berghei and other murine pathogens).

To address these problems, we aimed at developing an *in vivo* methodology for the prediction of optimal drug combinations employing a humanized mouse model of human malaria caused by Plasmodium falciparum. In this model, NODscidIL2Rγc^null^ (NSG) mice are engrafted with human erythrocytes (hE), thereby rendering mice susceptible to the P. falciparum strain 3D7^0087/N9^ (PfalcHuMouse model) (12). The PfalcHuMouse model lacks significant parasite sequestration in the vascular system or in organs (13), resulting in the absence of lethality with easy and precise quantitation of freely circulating mature parasites, unlike human studies, in which there is attachment of mature stages of P. falciparum to vascular endothelium (14, 15). Because of these advantages, the PfalcHuMouse model has been used to test the efficacy of drugs from numerous discovery programs in standardized dose-response studies (16–32) which have provided a rich pool of new drug candidates for combination (www.mmv.org).

In this work, we examined the robustness of the PfalcHuMouse model and the relative relevance of parasite clearance, recrudescence, and cure as variables of therapeutic response. These variables were used to investigate the individual contributions of antimalarial drugs in combination by using small experimental studies supported by historical data generated during early phases of discovery of the partner drugs.

## RESULTS

### Reproducibility of the PfalcHuMouse model.

**FIG 1:**
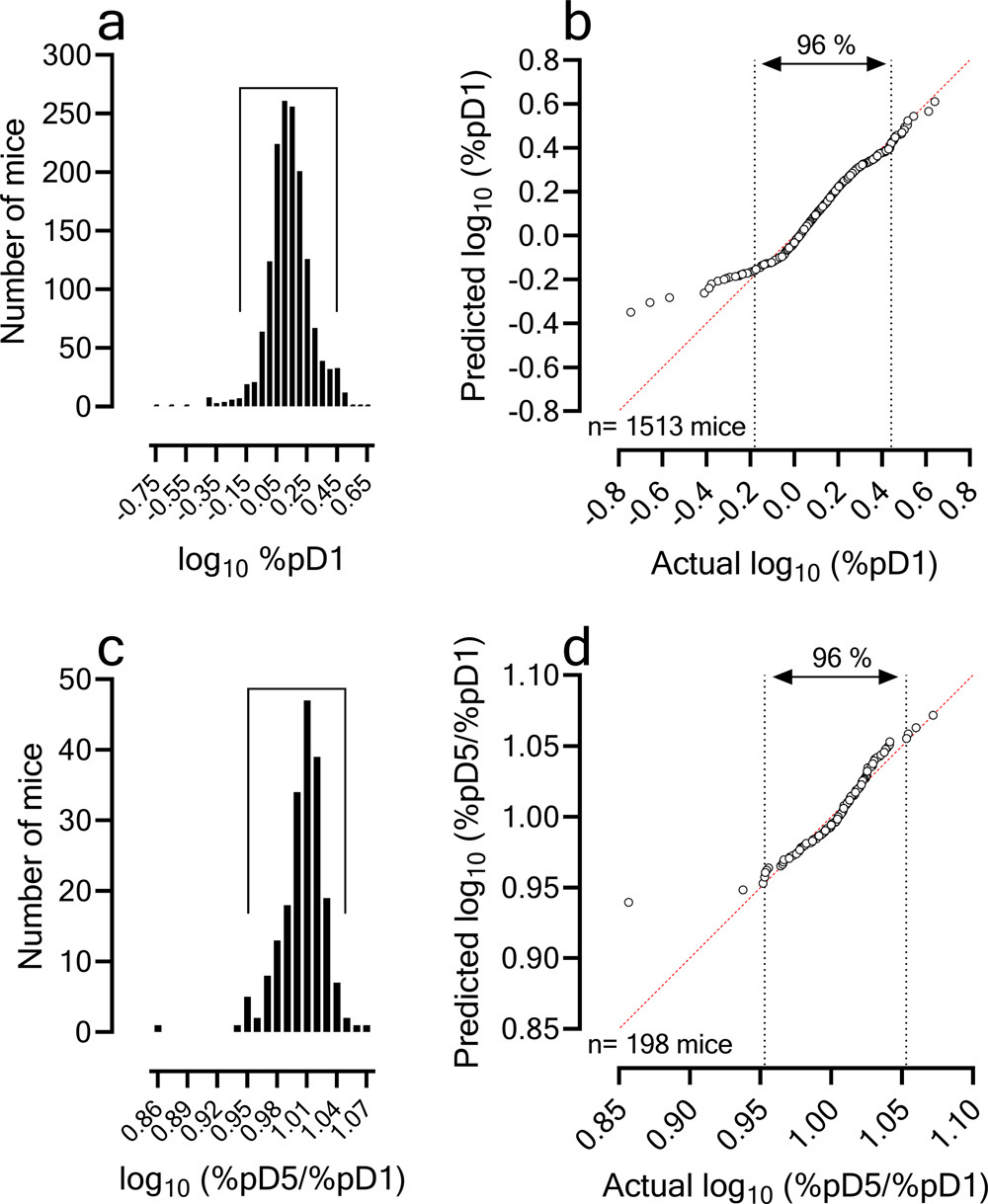
Reproducibility of the P. falciparum 3D7^0087/N9^ HuMouse model. (a) Distribution of the log_10_ (parasitemia at day 1, i.e., treatment start) in PfalcHuMice infected intravenously (i.v.) under standard conditions (see Materials and Methods). Data are historical data at TAD available at the time of reporting. The period marks the 2nd and 98th percentiles at −0.18 and 0.44, which encompassed 96% of data with a mean of 0.13 (corresponding to 1.35%). (b) Quantile-quantile (Q-Q) plot of normality for the variable log_10_ parasitemia at day 1 showing observed values versus expected normal values (*n*  =  1,513 mice). The vertical lines mark the 2nd and 98th percentiles as in panel a. (c) Distribution of the log_10_ ratio parasitemia at day 5/parasitemia at day 1. The period marks the 2nd and 98th percentiles at 0.95 and 1.05, which encompassed 96% of data. (d) Q-Q plot of normality for the variable log_10_ ratio parasitemia at day 5/parasitemia at day 1 showing observed values versus expected normal values (*n*  =  198 mice).

We examined the interassay reproducibility and robustness of the PfalcHuMouse model by analyzing historical data of its use in drug discovery projects other than drug combinations over the last 5 years. To assess reproducibility and robustness, we analyzed (i) the distribution of parasitemia at the onset of drug treatment and (ii) the rate of parasite growth over two asexual parasite cycles *in vivo*. First, the parasitemia at the onset of treatment was originally set up to match the parasite burden in humans as a function of body weight (i.e., parasite density). This normalization approach was chosen because body weight correlates with blood volume and has been used for allometric scaling of many physiological constants among mammals. Thus, the number of parasites in adult patients weighing 70 kg at hospital admission had been previously estimated to be 7 × 10^11^ parasites (33) (i.e., log_10_ parasites/kg_body weight_ = 10). In the PfalcHuMouse model, an inoculum of 30 × 10^6^ infected erythrocytes per mouse produced an average parasitemia of 1.42% (log-normal distribution; 95% confidence interval [CI_95%_], 1.40 to 1.45 for 1,513 mice, representing all mice that entered an experimental study during the period of reference) at treatment start (i.e., day 1 of study, 72 h after infection [Fig. 1a]). The average total erythrocyte concentration in blood of HuMice was 6.43 × 10^6^ erythrocytes/mL (CI_95%_, 6.38 to 6.48 erythrocytes/mL; data available for 963 mice). Thus, for an estimated volume of distribution of 1.5 mL and 25 g of body weight, the estimated average density of parasites in PfalcHuMice was 9.74 for log_10_ parasites/kg_body weight_, which was on the order of the human estimate. Interestingly, 96% of parasitemia determinations at day 1 of the study fell between 0.66 and 2.7%, making the log_10_ percent parasitemia on day 1 almost normally distributed, with a rate of 2% for unexpectedly low parasitemias (Fig. 1b).

Second, the growth rate of P. falciparum 3D7^0087/N9^ *in vivo* was measured as the ratio between parasitemia at day 5 (pDay5) and pDay1 in untreated control individuals. The mean log_10_ pDay5/pDay1 was 1.006 (*n* = 198 mice; CI_95%_, 1.002 to 1.009; coefficient of variation [CV], 2.35%) (Fig. 1c and d). This number of mice represented only those individuals used as controls of parasite proliferation (2 mice per study on average). Overall, the results indicated that the growth of P. falciparum 3D7^0087/N9^ in the humanized model was highly reproducible and the variability in parasitemia at drug treatment inception should be attributed to technical differences in parasite inoculation.

### Pharmacodynamic variables of therapeutic response.

**FIG 2:**
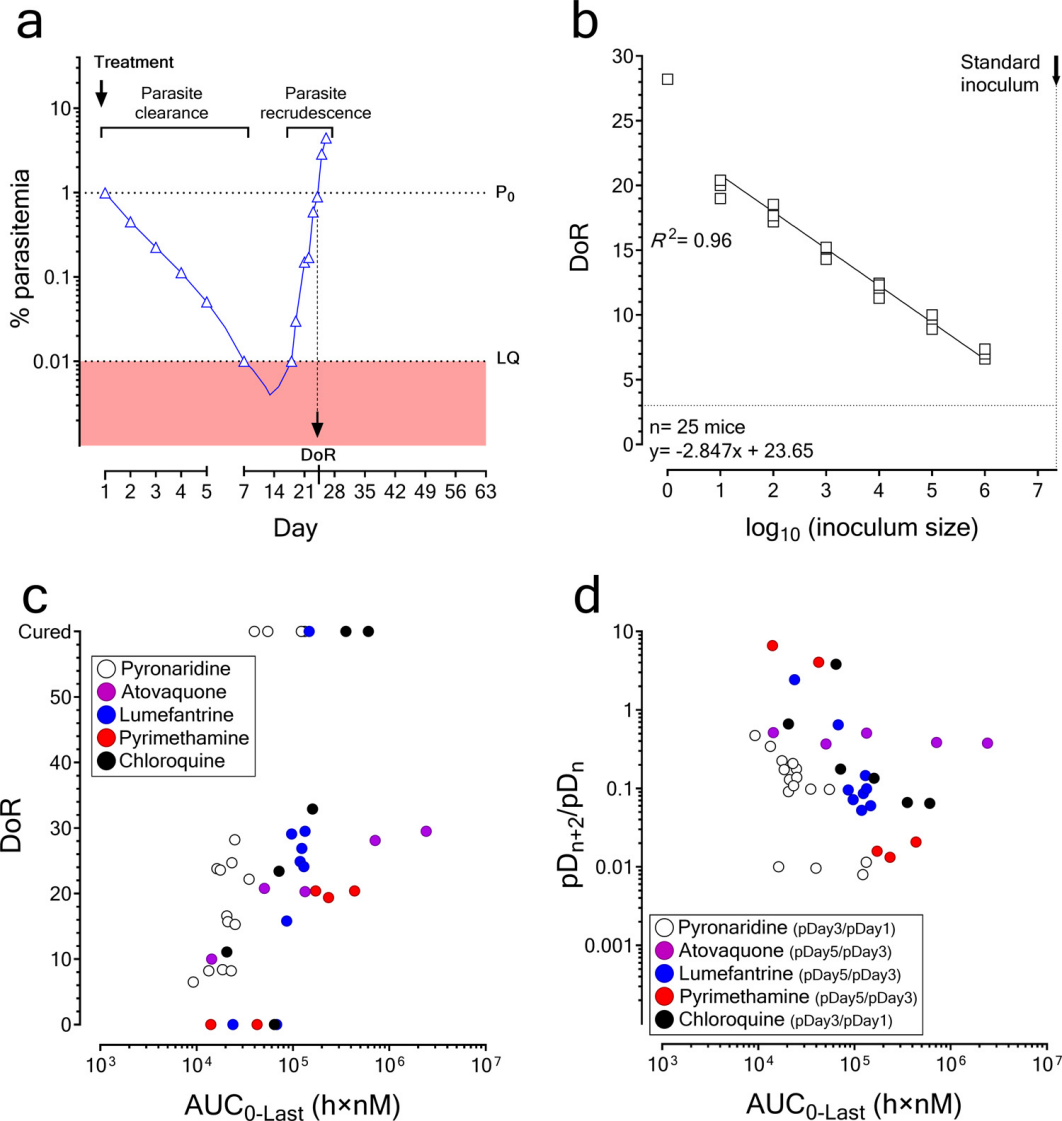
Variables of therapeutic response. (a) Suboptimal therapeutic response *in vivo*. The variables considered were PR (expressed as pD*_n_*_+2/_pD*_n_*), day of recrudescence (DoR), and cure (i.e., nondetectable parasitemia at day 60). (b) Log-linearity of DoR as a function of the inoculum size. PfalcHuMice were infected i.v. and DoR was calculated as the day at which the mean parasitemia at day 1 was achieved for each individual. (c and d) Distribution of DoR and pD*_n_*_+2/_pD*_n_* as a function of drug exposure in blood for standard antimalarials in monotherapy. Data were historical data of standard efficacy assays available at TAD at the time of reporting. HuMice reaching day 60 of assay with no detectable parasitemia in blood were considered cured.

To implement the PfalcHuMouse model for analysis of combinations, we first defined the variables of therapeutic response and their interpretation in the context of this analysis. These variables were parasite clearance (PC), parasite recrudescence (PR), and cure (Fig. 2a). PC depends on the mechanism of action of the drugs; it may affect both viable and nonviable parasites and is typically assessed by estimating the slope of the log-linear curve of parasitemia in peripheral blood (34). In our studies, we estimated PC by measuring the ratio of parasitemia in blood at 48-h intervals (pD*_n_*/pD*_n_*_+2_, where pD stands for parasitemia and n indicates the day of the assay), which was a simplified alternative of the classical approach and is frequently used in clinical studies. Conversely, PR depends on the number of viable parasites remaining in the body that could proliferate after noneffective treatment and is not systematically used to measure parasite killing *in vivo*. To test PR validity, we hypothesized that the time point for recrudescent parasitemia to reach the value at the onset of drug treatment (i.e., day of recrudescence [DoR]) correlated with the number of parasites that survived antimalarial treatment (35). In our system, DoR could range from day 0, when a drug failed to inhibit parasitemia, until day 60, at which individuals were deemed cured if no viable parasites were detected in peripheral blood. The selection of day 60 as endpoint of the study was based on theoretical (i.e., one parasite should lead to detectable parasitemia in less than 30 days) and practical (i.e., we have not observed any case of recrudescence beyond that day in more than 15 years of operation of the model) considerations. It is important to note that both DoRs, days 0 and 60, represented not quantifiable magnitudes but labels of specific conditions (i.e., therapeutic failure and cure, respectively).

DoR is not routinely used as a quantitative variable to assess parasite killing *in vivo*. To characterize this variable, we evaluated the linearity of DoR as an indicator of the number of viable parasites in the PfalcHuMouse model remaining after drug-mediated parasite clearance. To assess this contention without genetically modifying P. falciparum 3D7^0087/N9^ (e.g., using green fluorescent protein [GFP]-transgenic P. falciparum), we injected different numbers of P. falciparum-infected erythrocytes by the intravenous route and measured the DoR in mice as the time required to achieve a parasitemia of 1.42% in peripheral blood in the absence of treatment (Fig. 2b). We found that the DoR was a linear function of the log_10_ inoculum size over at least 6 logs (from 10^1^ to 3 × 10^7^). In this system, 1 log decrease in parasite numbers represented 2.85 days increase of DoR (data pooled from two independent studies; *n* = 25 mice, *R*^2^ = 0.96). Extrapolation of the regression line indicated that the expected DoR for a single inoculated parasite was 23.7 days, counted from the day of inoculation. Accordingly, the expected range of maximum DoR in standard efficacy studies encompassing 4 days of administration should be between 28 days for a rapidly parasiticidal drug and 35 for a slowly parasiticidal drug, counted from the day of last drug administration. However, the predictive power of the extrapolation of the DoR for a single parasite must be interpreted with caution because of the breakdown of linearity for inocula of ≤10 parasites, likely reflecting the stochastic nature of parasite growth from extremely low starting numbers.

To confirm this prediction, we analyzed historical data of the PfalcHuMouse model on the patterns of pD*_n_*_+2_/pD*_n_* and DoR for a set of oral antimalarials with known mechanism of action at different dose levels and schedules of administration (*n* = 41 mice). The drugs for which curative exposures were available in the data set, namely, chloroquine, lumefantrine, and pyronaridine, did not result in recrudescence after day 35, roughly consistent with the theoretical calculation described above (Fig. 2c). The analysis of individuals treated with other antimalarials was less conclusive because there were no cases of individuals cured after treatment available in The Art of Discovery’s (TAD’s) data set. These plots suggested that there was a functional relationship between drug exposure in blood and pD*_n_*_+2_/pD*_n_* or DoR for each individual mouse tested. It is noteworthy that the inspection of the profiles of parasitemia (Fig. 2c and d and Fig. S1) suggested that DoR had a wider dynamic range than pD*_n_*_+2_/pD*_n_* to detect antimalarial parasite killing effects of drugs than PC. Thus, DoR unveiled significant differences in parasite killing among individuals with the same PC, which is consistent with recently published data for the same model (36).

### Evaluation of ferroquine-artefenomel and piperaquine-artefenomel.

Next, we tested the combinations artefenomel (OZ439) plus ferroquine and artefenomel plus piperaquine, which have been recently evaluated in clinical trials (37, 38), as proof of concept to assess the relative relevance of PC, DoR, and cure as variables of therapeutic response in the PfalcHuMouse model.

To simplify the study of these combinations, we only considered drug exposure in blood (area under the curve for ferroquine [AUC_ferroquine_], AUC_piperaquine_, and AUC_artefenomel_) as a PK explanatory variable instead of the dose administered to PfalcHuMice, which is severely affected by the vehicles of administration (39), the physical properties of the specific batches of drug employed in the studies (40), and the individual variability of HuMice. As in the clinical study, for ferroquine, we only considered the concentration of the parent compound to estimate the AUC_ferroquine_ because the concentration of SSR97213, the main metabolite of ferroquine, represented an average of 40% of the total exposure of ferroquine plus SSR97213 and would not add information to the statistical analysis of data (37). Using this approach, each individual had associated values for drug exposure in blood (PK explanatory variables) and for individual therapeutic response (i.e., PR, DoR, and cure), all of which were measured experimentally. Therefore, we reduced the complexity of time-dependent measurements of parasitemia and PK pertaining to a single individual (see Fig. S1 to S9 in the supplemental material) to a set of five real numbers (i.e., a five-component vector). Thus, each vector represented an experimental sample of the functional relationship between the therapeutic response and pharmacological treatment.

Given their physicochemical properties, both ferroquine and piperaquine might accumulate in infected erythrocytes (41), so we mined data on the distribution of these drugs in plasma, erythrocytes, and infected erythrocytes of the PfalcHuMouse from previous blood partitioning studies in unrelated drug discovery programs. Approximately 97.5% of both ferroquine and piperaquine was associated with circulating erythrocytes, while only 2.5% was found in plasma. For piperaquine, the accumulation was even higher in erythrocytes of infected individuals (>99%). Moreover, as previously reported (13), there was no sequestration of P. falciparum in vascular endothelium of the PfalcHuMouse. Therefore, the contribution of drug accumulated in mature parasites circulating in blood might be relevant, especially in proliferating parasites exposed to noninhibitory concentrations. Consequently, we corrected this effect on drug exposure in blood by dividing the total drug exposure levels in blood by the number of infected erythrocytes in circulation for each animal in the study.

**FIG 3:**
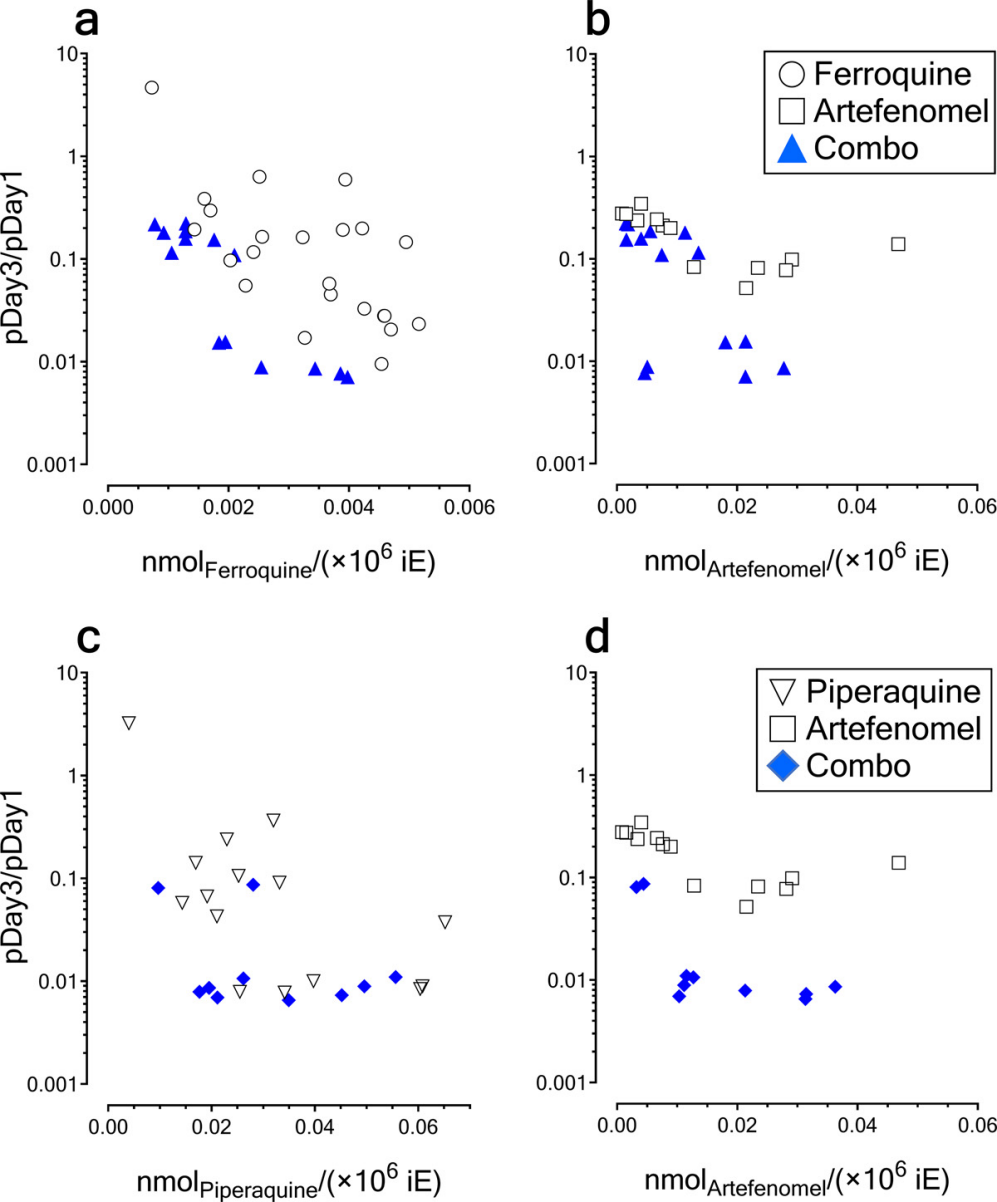
Analysis of parasite clearance in combinations in the P. falciparum 3D7^0087/N9^ HuMouse model. The pairs of plots in panels a and b and panels c and d represent orthogonal views on three-dimensional plots of drug efficacy for the variables pDay3/pDay1 (i.e., parasite clearance) and drug exposure from treatment inception to 48 h in blood of HuMice (expressed in nmol_drug_/10^6^ iE) for ferroquine plus artefenomel (a and b) and piperaquine plus artefenomel (c and d), respectively.

When we compared PC of monotherapies and combinations, we observed a trend toward faster parasite elimination in the combinations in a very narrow range of the lowest exposures of partner drugs, particularly for the combination ferroquine plus artefenomel (Fig. 3). Of note, in Fig. 3, a lower ratio pDay3/pDay1 represented a faster clearance of parasites. However, at higher exposures, parasite clearance might be explained by ferroquine and piperaquine, respectively.

**FIG 4:**
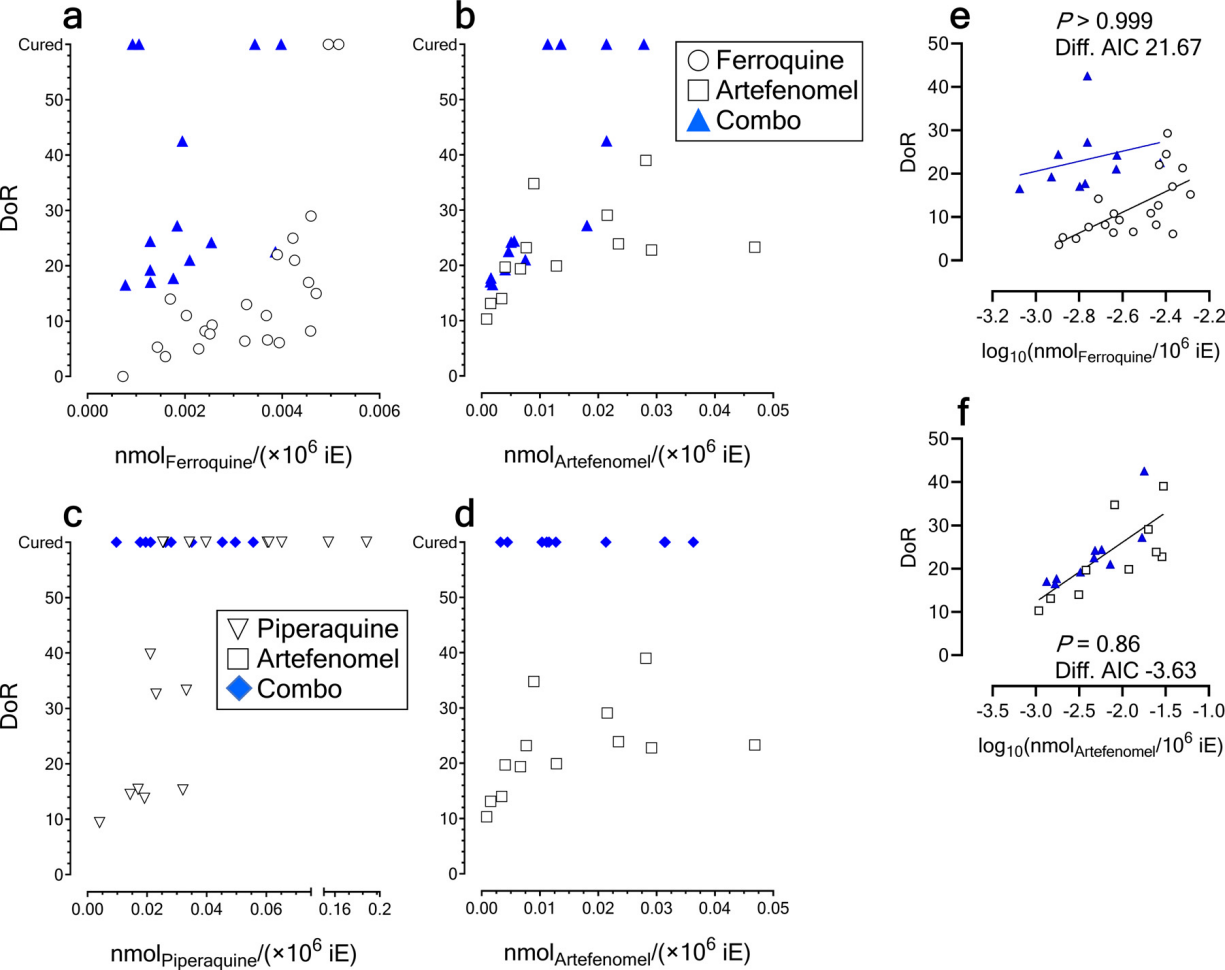
Analysis of DoR as a function of drug exposure in blood of the P. falciparum 3D7^0087/N9^ HuMouse model. The pairs of plots in panels a and b and panels c and d represent orthogonal views on three-dimensional plots of the variable DoR and drug exposure in blood of HuMice (expressed in nmol_drug_/10^6^ iE) for ferroquine plus artefenomel (a and b) and piperaquine plus artefenomel (c and d), respectively. (e) Comparison of DoR for individuals treated with ferroquine or ferroquine plus artefenomel. The plot shows the probability of the two sets having different regression lines according to the Akaike information criterion. Only individuals for which DoR was not 0 or had recrudescent parasites detected in peripheral before day 60 were analyzed. (f) Comparison of DoR for individuals treated with artefenomel or ferroquine plus artefenomel. The plot shows the probability of the two sets sharing the same regression line according to the Akaike information criterion. Only individuals for which DoR was not 0 or had recrudescent parasites detected in peripheral blood before day 60 were analyzed.

Next, we compared the DoR of individuals treated with the combination to individuals treated with monotherapy by multivariate linear regression. The individuals for which drug treatment did not have any effect (i.e., DoR = 0) and those deemed cured (i.e., individuals with no detectable recrudescent parasitemia at day 60) were excluded from the analysis because their actual DoR was indeterminate, as pointed out in the previous section. The data shown in Fig. 4a, b, e, and f suggested that all the effect on DoR after treatment with ferroquine plus artefenomel could be explained by the contribution of artefenomel alone when DoRs of 0 or 60 were excluded. That is, the individuals having similar exposures of artefenomel in blood tended to have similar DoRs irrespective of their previous treatment with artefenomel monotherapy or the combination with ferroquine.

In the case of the combination piperaquine plus artefenomel, a quantitative comparison with their respective monotherapies of their effect on DoR was not possible at all because all individuals treated with the combination were cured over the range of exposures in which piperaquine and artefenomel monotherapies were not curative (Fig. 4c and d). Therefore, DoR was insufficient for a complete statistical analysis of drug contributions to the combination, even though the data strongly suggested some degree of enhancement of parasite killing.

### Analysis of cure.

To overcome the limitations of DoR observed in our studies, we addressed the analysis of cure of individuals as a function of drug exposures in blood by logistic regression because this technique would allow inclusion of data from individuals excluded from DoR analysis. The analysis of pooled historical data on monotherapies and experimental data on combinations by logistic regression (Table S1) showed that the therapeutic effect of ferroquine and artefenomel was best explained by the sum of the effect size of the exposure of each drug according to the equation

$$\text{log}(\frac{p}{1-p})=17.2+5.4\times {\text{log}}_{10}{\text{AUC}}_{\text{ferroquine}}+1.6\times {\text{log}}_{10}{\text{AUC}}_{\text{artefenomel}}$$

(sample size, *n* = 50; Tjur’s *R*^2^ = 0.31), which was qualitatively consistent with findings in clinical trials (37).

A significant improvement on the coefficient of determination could be obtained by considering an interaction term between drug exposures in the logistic model. However, we observed a high degree of correlation among the explanatory variables (variance inflation factor (VIF) higher than 10 for all explanatory variables). Therefore, the data set did not provide convincing evidence to include such interaction term in the logistic model. To further explore the possibility of the existence of a term of interaction, we examined the effect of adding a virtual individual cured in monotherapy with artefenomel at plausible exposure levels on the logistic function of probability. The results consistently showed that including a factor of mechanistic interaction between the drugs would be preferable over a simple model of summation of individual drug effects (39.1% probability of a model without a term of interaction versus 68.9% probability of a model with a term of interaction, Akaike information criterion). Therefore, cure analysis predicted some level of mechanistic interaction between ferroquine and artefenomel that was not apparent in the analysis of DoR by multiple linear regression, likely due to discarding individuals not eligible for the last type of analysis.

The logistic analysis of artefenomel plus piperaquine supported the notion that both artefenomel and piperaquine significantly contributed to cure of mice according to the equation

$$\log(\frac{p}{1-p})=102.8+24.2\times {\text{log}}_{10}{\text{AUC}}_{\text{piperaquine}}+16.2\times {\text{log}}_{10}{\text{AUC}}_{\text{artefenomel}}$$

(sample size, *n* = 40 individuals; Tjur’s *R*^2^ = 0.91). Modeling the logistic function by including virtual individuals cured with artefenomel monotherapy at plausible exposures and balanced numbers predicted that the contributions of the two drugs would be similar. In this combination, the introduction of a term of interaction led to no convergence of the logistic model because there was no overlap between the sets of cured and noncured individuals (i.e., drug exposure allowed a perfect separation between sets of cured and noncured individuals). This result supported the possibility of a mechanistic interaction among the drugs as well, which was not detected in the corresponding clinical study (38).

**FIG 5:**
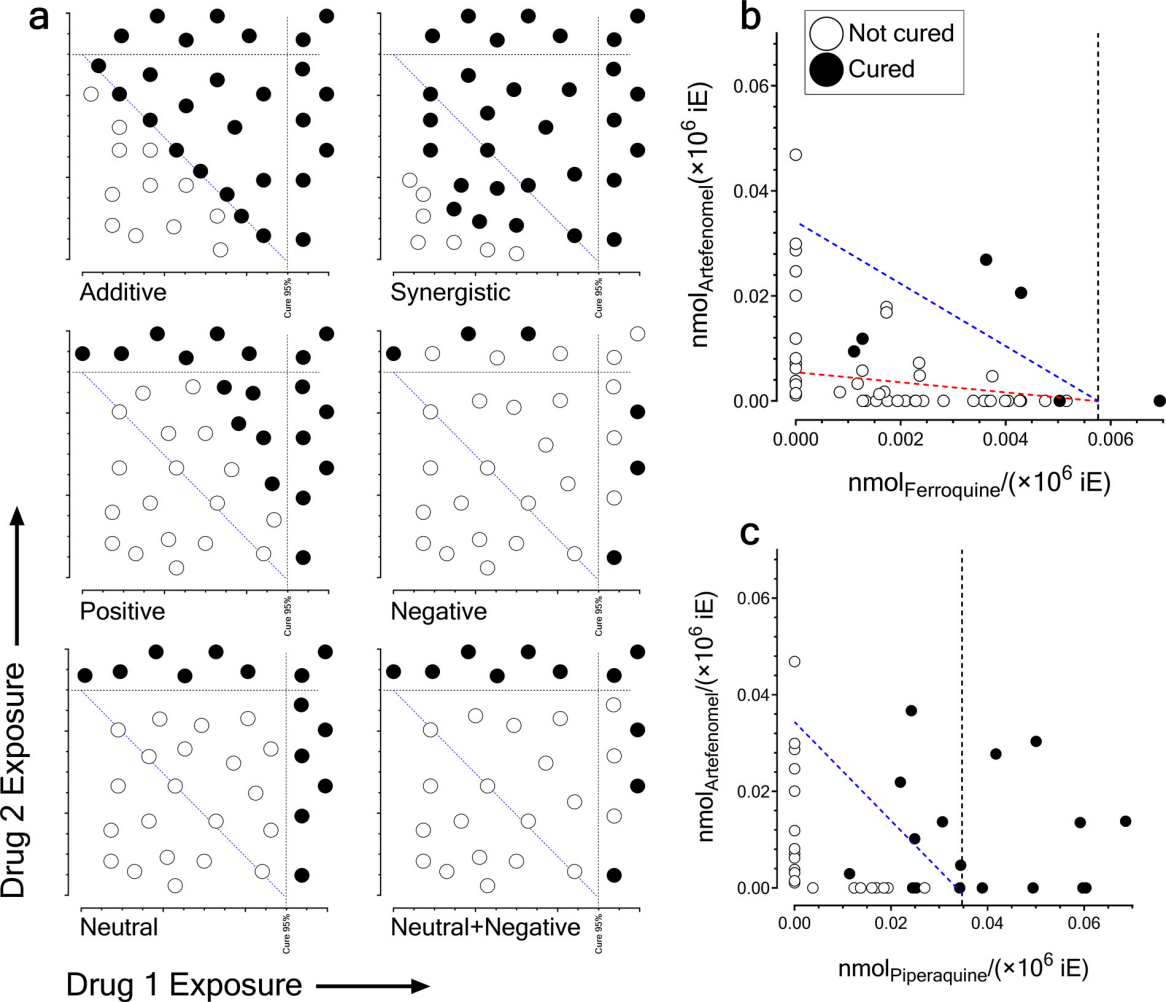
(a) Theoretical patterns of cure of P. falciparum 3D7^0087/N9^-infected HuMice as a function of drug exposure assuming equipotency of drugs. The plots show idealized distributions of cured and noncured individuals as a function of drug exposure in blood. The lines orthogonal to the *x* and *y* axes represent the exposure associated with a 95% probability of cure in monotherapy. The blue dashed line represents the set of points of theoretical functional complementarity between the drugs for equipotent drugs. (b) Pattern of cure of P. falciparum 3D7^0087/N9^-infected HuMice treated with ferroquine plus artefenomel. The dashed lines represent the points of theoretical complementarity at theoretical equipotency (red dashed line) or according to realistic estimates of potency for artefenomel (blue dashed line) to achieve 95% probability of cure. The line orthogonal to the *x* axis represents the exposure associated with a 95% probability of cure in monotherapy. (c) Pattern of cure of P. falciparum 3D7^0087/N9^-infected HuMice treated with piperaquine plus artefenomel. The blue dashed line represents the set of points of theoretical functional complementarity between the drugs assuming equipotency of artefenomel and piperaquine. The line orthogonal to the *x* axis represents the exposure associated with a 95% probability of cure in monotherapy.

Next, we built a graphical tool to further investigate the type of mechanistic interactions among drugs in an intuitive way by testing a very limited number of individuals in experimental combination studies *in vivo* to minimize animal usage. The tool was established as a bidimensional plot relating the exposure of each drug and the cure of individuals leading to 95% cures in PfalcHuMice, which is a quantitative criterion widely accepted in clinical trials for successful treatments (42). The plot would display the lines of exposure at 95% cure in monotherapy, and a line connecting the points of curative exposure of a drug in the absence of the corresponding partner drug (Fig. 5a). This connecting line would represent the line of theoretical complementarity, that is, where the sum of exposures that would lead to 95% probability of cure in the presence of both drugs. According to this approach, we distinguished six general patterns of interaction, based on the distribution of cures of a small number of individuals tested in combination, and compared those with the combination data sets (Fig. 5b and c). Using historical data available at TAD, the exposures of ferroquine and piperaquine at 0.58 × 10^−3^ and 3.47 × 10^−3 ^nmol/10^6^ infected erythrocytes (iE), respectively, led to 95% probability of cure (logistic regression; *n* = 23 individuals and Tjur’s *R*^2^ = 0.64 for ferroquine; *n* = 17 individuals and Tjur’s *R*^2^ = 0.80 for piperaquine). No cures were available at TAD’s database for monotherapy with artefenomel up to 4.7 × 10^−3 ^nmol/10^6^ iE with the schedules of treatment used in the experimental studies (Fig. S9). The graphic analysis suggested that artefenomel was at least 10-fold less potent than ferroquine for killing P. falciparum parasites *in vivo* (Fig. 5b) and their mechanistic pattern of interaction might be additivity conditioned by their large difference in intrinsic potency. In contrast, assuming that the potency of artefenomel for killing P. falciparum *in vivo* were in the same order of magnitude of piperaquine, as a worst-case scenario, their pattern of interaction suggested that these drugs might be synergistic on parasite killing *in vivo* (Fig. 5c).

Overall, the analysis of cure made possible the use of all experimental data available, allowing the direct estimation of the individual contributions of combined drugs by using small subsets of experimental data of combinations analyzed with larger data sets of historical data available from the PfalcHuMouse model.

## DISCUSSION

In this study, we explored the use of the PfalcHuMouse model to identify optimal combinations of antimalarials *in vivo*. Our results showed that the model is robust and amenable for studies addressing mechanistic antiparasiticidal interactions of candidate drugs by using small cohorts of mice treated with combinations and historical data of the partner drugs in monotherapy.

Interestingly, pathogen clearance from peripheral blood was a sensitive predictor neither of disease outcome nor of the mechanistic drug interaction. Conversely, the analysis of the remaining viable parasites in blood by assessing recrudescence and cure were both informative and complementary. This finding is in line with recent publications stressing the value of viability to assess the effect of drugs in volunteer infection studies (VIS) (43, 44).

The definition and characterization of recrudescence (i.e., DoR) as a variable of therapeutic response presented in this paper represents a novelty with respect to the usual statistical treatment of this variable. In general, recrudescence has been traditionally viewed as a “time-to-event” variable (i.e., time until first detection in peripheral blood above the limit of quantification) amenable for survival analysis techniques (45). In this work, we showed that DoR can also be treated as a continuous variable based on the kinetics of growth of P. falciparum 3D7^0087/N9^. This fact markedly enhanced the predictive power to modeling the effect of drugs *in vivo*. Of note, in practice, our definition of DoR is independent of the limit of quantification of the technique used to measure parasitemia, conversely to the traditional time of parasite first appearance in blood, which critically depends on that limit. In our model implementation, the level of parasitemia at P_0_ (see Materials and Methods) is in the optimal range of accuracy, sensitivity, and specificity of the flow cytometry technique (46). However, the measurements of parasitemia at the time of first parasite detection are intrinsically less accurate because measurements are made close to the limit of quantification of flow cytometry. Despite such advantages, the assessment of DoR was limited by the loss of relevant data out of the boundaries of DoR definition that were clearly revealed in this study. Less obvious limitations of the use of DoR are associated with the variability of the growth kinetics *in vivo* that different strains of P. falciparum might have and/or changes in that kinetics caused by immunomodulatory interventions in the humanized model (47). Therefore, in its present form, DoR seems a powerful tool to analyze and interpret the parasitological effects of drugs within the limits of a defined *in vivo* model rather than allowing a direct comparison among different models.

Assessment of cure was technically and analytically simpler and more powerful than the assessment of DoR and overcame the limitation of the latter for the comparison of susceptibility of P. falciparum strains mentioned above. This technical characteristic of cure analysis opens the possibility of extending this analysis to PfalcHuMice infected with field isolates (48). These new extended models might be used to explore experimentally the genetic plasticity and complex biology of *Plasmodium* species in the field, which is a major risk for implementing new interventions to eradicate malaria. Thus, it seems conceivable that these expanded PfalcHuMouse models might be used to investigate what the effect of phenotypes and genotypes of field isolates on the efficacious ratios of drugs tested as fixed combinations could be. Hopefully, this might help predicting efficacious exposures to prevent treatment failures in clinical trials in countries of endemicity (37, 38). Supporting this approach, we estimated that the average exposure of artefenomel achieved in clinical trials of combinations of artefenomel plus ferroquine (37) and artefenomel plus piperaquine (38) was about 0.02 nmol/10^6^ iE, which would have been predicted not to be curative against field parasites of comparable sensitivity to the drug-sensitive P. falciparum 3D7^0087/N9^ strain. On the contrary, ferroquine and piperaquine exposures were in the range of 100- to 1,000-fold and 1- to 10-fold higher in patients than their respective 95% curative exposures in the PfalcHuMouse model. Moreover, the logistic analysis in the clinical studies showed that the odds for cure were a function of the concentration in plasma of the drugs at day 7 after treatment, the parasite burden at treatment inception, and the geographical region where the studies were conducted. Interestingly, in both cases artefenomel was found to have a higher impact on the odds of cure than ferroquine or piperaquine. However, the opposite was found in the PfalcHuMouse model by using the same statistical approach but employing a drug-sensitive P. falciparum strain at a homogeneous parasite density at treatment inception. Therefore, despite the technical uncertainties in the methodologies and objective conditions to extrapolate experimental results from PfalcHuMice to clinical results, it is tempting to speculate that failures of artefenomel plus ferroquine or piperaquine in humans might have been due to suboptimal exposure of artefenomel in a context of field isolates with reduced sensitivity to ferroquine and piperaquine as an explanation for the unsuccessful clinical trials. The extended PfalcHuMouse models might well be used to test experimentally this hypothesis by using HuMice infected with adapted *Plasmodium* field isolates from the target human populations or even employing isolates obtained from treated patients in whom treatment failed and recrudescent parasites were collected. These possibilities suggest that the PfalcHuMouse model might be expanded to integrate strains of P. falciparum representing the susceptibility of field parasites to produce realistic estimates of efficacious exposures of combinations. This approach may have some intrinsic limitations derived from the process of P. falciparum strain adaptation and the physiological differences between humans and HuMice. Concerning the first limitation, two major potential risks of the approach are that some clinical field isolates might not be adapted to HuMice and that the process of adaptation might dramatically change the pattern of susceptibility to drugs with respect to the native strains. The physiological differences between hosts, humans, and HuMice may impose different environmental conditions on parasites, thereby conditioning their repertoire of responses to drugs. In all cases, there might be a risk of introducing some systematic bias on experimental modeling of the estimates of efficacious exposures. Against these risks, detailed studies of the process of adaptation to HuMice have shown that the genotypic changes are associated with the modification of parasite surface antigens expressed on infected erythrocytes to avoid macrophage phagocytosis and not with point mutations in specific genes involved in metabolism (49). Despite these encouraging results, it would seem reasonable to estimate the actual impact of the two aforementioned intrinsic limitations for predictive modeling by addressing a proof-of-concept/validation study testing a limited number of clinical strains of known properties for adaptation to HuMice and their susceptibility to standard drugs.

Combinations are used to protect drugs against the selection of resistant parasites and/or to reduce toxicity in the host. Although the use of the PfalcHuMouse model for toxicological assessments is questionable, in this model the frequency of resistance is directly addressed by measuring cure and addressing genetic analysis of recrudescent parasites. Thus, as the numbers of parasitized erythrocytes can be easily estimated for PfalcHuMice, each mouse could be interpreted as an independent test point of the relationship among drug exposures, the frequency of resistance in the P. falciparum strain, and the appearance/selection of specific unpredictable mutations in the genome of the parasite. This would include searching for single nucleotide polymorphisms and gene copy number variations after whole-genome sequence analysis of recrudescent and parental parasites as relevant explanatory covariates (50). The simplicity of the experimental outcome and robust statistical modeling would allow the systematic investigation and modeling of genetic, epigenetic, and biochemical factors that promote the survival of field strains to drug combinations. Interestingly, the threshold for treatment of 10^8^ parasites could be raised to 10^9^ per mouse. These conditions would match parasite densities of *circa* 10^12^ in humans. Addressing mutation frequencies of 10^−10^ could be achieved with cohorts of PfalcHuMice representing conditions of heavy parasite burdens in patients. These considerations may have relevance for the experimental modeling of clinical circumstances in which resistance develops. These include the high consumption of antimalarials for the treatment of fever of unknown origin in countries of endemicity (51). In this case, significant numbers of individuals carry residual concentrations of drugs over long periods, allowing for the selection and spread of drug-tolerant parasites. Our results suggest that the PfalcHuMouse model might be used not only to assess the parasite propensity to develop antimalarial resistance (44, 48) but also to identify possible series of intermediate drug-tolerant mutations and help to understand the evolution and spread of resistance (52), because all stages of the parasite are accessible to analysis. On the other hand, the lack of parasite sequestration and adaptive immune system in the PfalcHuMouse model, although convenient as an investigational tool in drug discovery, might introduce some systematic bias in the selection of resistant variants. Specifically, sequestered parasites in organs of human patients may be related to parasite selection pressure by exclusion from or overexposure to drug treatments, whereas these situations are unlikely to happen in the PfalcHuMouse model. Similarly, the adaptive immune system is a strong selection pressure on the generation of drug-resistant parasites in humans (53). However, this pressure is absent in the PfalcHuMouse model. So, to what extent there would be a significant overlap between the space of mutations accessible to the parasite in *in vitro* assays (54), in *in vivo* tests in the PfalcHuMice (55), and in the field is an open question whose answer might help to better understand the environmental factors that affect the mechanisms of resistance of P. falciparum.

In summary, our results showed that the analysis of cure after treatment with drug combinations in the PfalcHuMouse model was the most informative technique for studying mechanistic antiparasiticidal interactions of candidate drugs *in vivo*. The cure analysis in the PfalcHuMouse model was amenable for studies using small cohorts of mice and historical data of the partner drugs in monotherapy. The data presented here also suggested that the PfalcHuEryMouse model might enable researchers to empirically interrogate, in a common methodological framework, the ratios and absolute concentrations of partner drugs in fixed combinations and assess combinations of immune-boosting drugs with one or more antiparasitic drugs, triple combinations for single-encounter radical cure and prophylaxis (SERCaP), and human monoclonal antibodies (MAbs).

## MATERIALS AND METHODS

### Ethics statements.

Studies with mice were performed at The Art of Discovery and approved by The Art of Discovery Institutional Animal Care and Use Committee (TAD-IACUC). This committee is certified by the Biscay County Government (Bizkaiko Foru Aldundia, Basque Country, Spain) to evaluate animal research projects from Spanish institutions according to point 43.3 from Royal Decree 53/2013, from 1 February (BOE-A-2013-1337). All experiments were carried out in accordance with European Directive 2010/63/EU. The results from the animal experiments are reported following ARRIVE guidelines except for disclosure of business trade confidential information (https://www.nc3rs.org.uk/arrive-guidelines). The human biological samples were sourced ethically, and their research use was in accord with the terms of the informed consents.

### Mice.

Pathogen-free immunodeficient inbred female NSG (NOD.Cg-PrkdcscidIl2rgtm1WjI/SzJ) mice were purchased from Charles River Laboratories (l’Arbresle, France) and adapted to the TAD (Derio, Basque Country, Spain) animal facility for at least 1 week before entering experimental procedures. The mice were used in experimental studies when their weight was in the range of 23 to 30 g. The mice were provided with ultrafiltered water (Innovive, San Diego, CA) and gamma-irradiated standard pelleted diet (Envigo, Indianapolis, IN) *ad libitum*.

### Parasite.

P. falciparum 3D7^0087/N9^ has been described previously (13). The strain was kindly donated by Sergio Wittlin (Swiss Tropical and Public Health Institute, Basel, Switzerland). A standard screening collection of frozen 3D7^0087/N9^ was produced at TAD by expansion in human erythrocyte (hE)-engrafted NSG mice. P. falciparum 3D7^0087/N9^ was maintained *in vivo* by serial passages to avoid the loss of competence to grow in humanized mice after *in vitro* culture.

### Human blood.

Concentrates of hE from malaria-negative donors were generously provided by the Basque Biobank (www.biobancovasco.org; Galdakao, Basque Country, Spain), Centro de Transfusiones de la Comunidad de Castilla y León (Valladolid, Spain), and Banc de Sang I Teixits (Barcelona, Spain). Blood samples were processed following standard operation procedures with appropriate approval of the Ethical and Scientific Committees. Blood was aliquoted and stored at 4°C until use. Prior to injection, hE were washed twice with RPMI 1640 and 25 mM HEPES (Sigma-Aldrich, St. Louis, MO) containing 7 × 10^−3 ^mM hypoxanthine (Sigma-Aldrich, St. Louis, MO) at room temperature. Finally, hE were resuspended at 50% or 75% hematocrit in RPMI 1640, 25% decomplemented human serum (Sigma-Aldrich), and 3.1 mM hypoxanthine and warmed at 37°C for 20 min prior to injection in mice.

### Infection.

Experimental infections were carried out as previously described (12), with minor modifications. Briefly, NSG mice were engrafted with hE by intraperitoneal daily injections of 1-mL hE suspensions until a minimum of 40% chimerism in peripheral blood was achieved (typically10 days of injections). The hE-engrafted mice (HuMice) were infected with 0.3 mL of a suspension of pooled blood collected by aseptic cardiac puncture from CO_2_-euthanized donor HuMice harboring a parasitemia of 5 to 10% P. falciparum 3D7^0087/N9^. The blood was diluted with saline at room temperature to obtain a single inoculum suspension per study at a concentration of 1.17 × 10^8^ parasitized hE/mL and injected in recipient HuMice through the tail lateral vein.

### Drug treatment.

Lumefantrine (AK Scientific, Union City, CA), chloroquine (Sigma-Aldrich, St. Louis, MO), pyrimethamine (AK Scientific), atovaquone (Sigma-Aldrich), and ferroquine (Medicines for Malaria Venture) were formulated in 1% methylcellulose (Acros Organics, Morris Plains, NJ) and 0.1% Tween 80 (Thermo Fisher Scientific, Fair Lawn, NJ) in double-distilled water. Piperaquine (AK Scientific) and artefenomel (Medicines for Malaria Venture) were formulated in 0.1% hydroxypropyl-methylcellulose (Sigma-Aldrich), 0.5% benzyl alcohol (Sigma-Aldrich), 0.4% Tween 80, 0.9% sodium chloride (Fisher Scientific, Loughborough, LE, UK) in double-distilled water. In some studies, piperaquine was formulated in double-distilled water. Pyronaridine (Medicines for Malaria Venture) was formulated in 0.5% hydroxypropyl-methylcellulose and 0.1% Tween 80.

Before drug administration, each infected mouse was randomly assigned to its corresponding treatment. Drug treatment started when infected mice had ~1.4% patent parasitemia in peripheral blood (day 1 of study, designated P_0_). The treatments were administered by oral gavage with 20-gauge straight reusable feeding needles (Fine Science Tools GmbH, Heidelberg, Germany). The volume of administration for the *per os* (p.o.) route was 10 mL/kg of body weight unless otherwise stated.

### Measurement of concentration of drugs in blood.

The concentrations of drugs were measured in whole blood of P. falciparum 3D7^0087/N9^-infected HuMice since the amount of plasma that could be obtained from each mouse was very low. Samples of peripheral blood (25 μL) were taken at different time points after dosing, mixed with 25 μL of 10.9 mM potassium oxalate and 59.5 mM sodium fluoride in Milli-Q water, and immediately frozen on a thermal block at −80°C. The frozen samples were stored at −80°C until analysis. Blood from P. falciparum 3D7^0087/N9^-infected HuMice harboring a parasitemia of ~1.4% was used for preparation of standard curves, calibration, and quality control samples. The drugs were extracted from 10 μL of lysates, obtained by protein precipitation of blood samples, by using standard liquid-liquid extraction methods. The samples were analyzed by liquid chromatography-tandem mass spectrometry (LC-MS/MS) for quantification in Waters Micromass ultrahigh-performance triple quadrupole (UPLC-TQD) (Waters, Manchester, UK) mass spectrometers.

### Measurement of parasitemia.

The quantification of parasitemia at different time points after the infection was performed by flow cytometry as previously described (46). Briefly, 2 μL of blood was taken from the lateral tail vein of HuMice and stained with SYTO-16 (Fisher Scientific), a membrane-permeable nonselective fluorescent nucleic acid dye to detect intraerythrocytic parasites, and TER119-phycoerythrin monoclonal antibody (Miltenyi Biotec, Bergisch Gladbach, Germany) as a marker of murine erythrocytes, exactly as described. The samples were acquired and analyzed in a Attune NxT acoustic focusing flow cytometer (Thermo Fisher Scientific, Waltham, MA). The limit of quantification was set to 0.01%, where a minimum of 100 infected events was considered the minimal statistically significant sample (~10^6^ total erythrocytes counted for detection). Parasitemia was expressed as the percentage of parasitized erythrocytes with respect to the total erythrocytes in circulation and/or as the absolute concentration of circulating parasitized erythrocytes. For qualitative assessment of blood samples from infected mice, blood smears stained with 10% (vol/vol) Giemsa in saline buffer (0.015 M NaCl, 0.001 M phosphate buffer [pH 7.0]) were prepared and analyzed as described previously (56) in a Zeiss Primo Star 4 microscope (Thermo Fisher Scientific) at a magnification of ×100.

The treated mice that reached the limit of detection by standard flow cytometry (<0.01% from total circulating erythrocytes) after pharmacological treatment were maintained until day 60 of study. Blood samples were regularly taken every 2 to 3 days and tested from the presence of circulating parasites.

### Data analysis.

Multivariate linear regression, logistic regression, and descriptive statistics were carried out using GraphPad Prism 9.0 (GraphPad Software, San Diego, CA). The comparison of regression models was executed using the Akaike information criterion (57). Probability values of <0.05 were considered significant. Phoenix WinNonlin 8.2 (Certara, St. Louis, MO) was used for PK standard analysis.

The drug exposure as a function of parasitemia was calculated as the ratio between the AUC_0 to last_ of the drug in blood (h × nmol/mL) divided by AUC_0 to 96 h_ of the curve of the concentration of parasites in peripheral blood (h × 10^6^ infected hE/mL).

### Data and material availability.

All unique materials are available upon reasonable request.
