# Supplementary material for: Predicting Optimal Antimalarial Drug Combinations from a Standardized Plasmodium falciparum Humanized Mouse Model
Source: Antimicrob Agents Chemother. 2023 May 3;67(6):e01574-22. doi: 10.1128/aac.01574-22 (PMC10269072; doi:10.1128/aac.01574-22)
Supplement: Supplemental file 1 — Supplemental material. Download aac.01574-22-s0001.pdf, PDF file, 6.6 MB [file aac.01574-22-s0001.pdf]

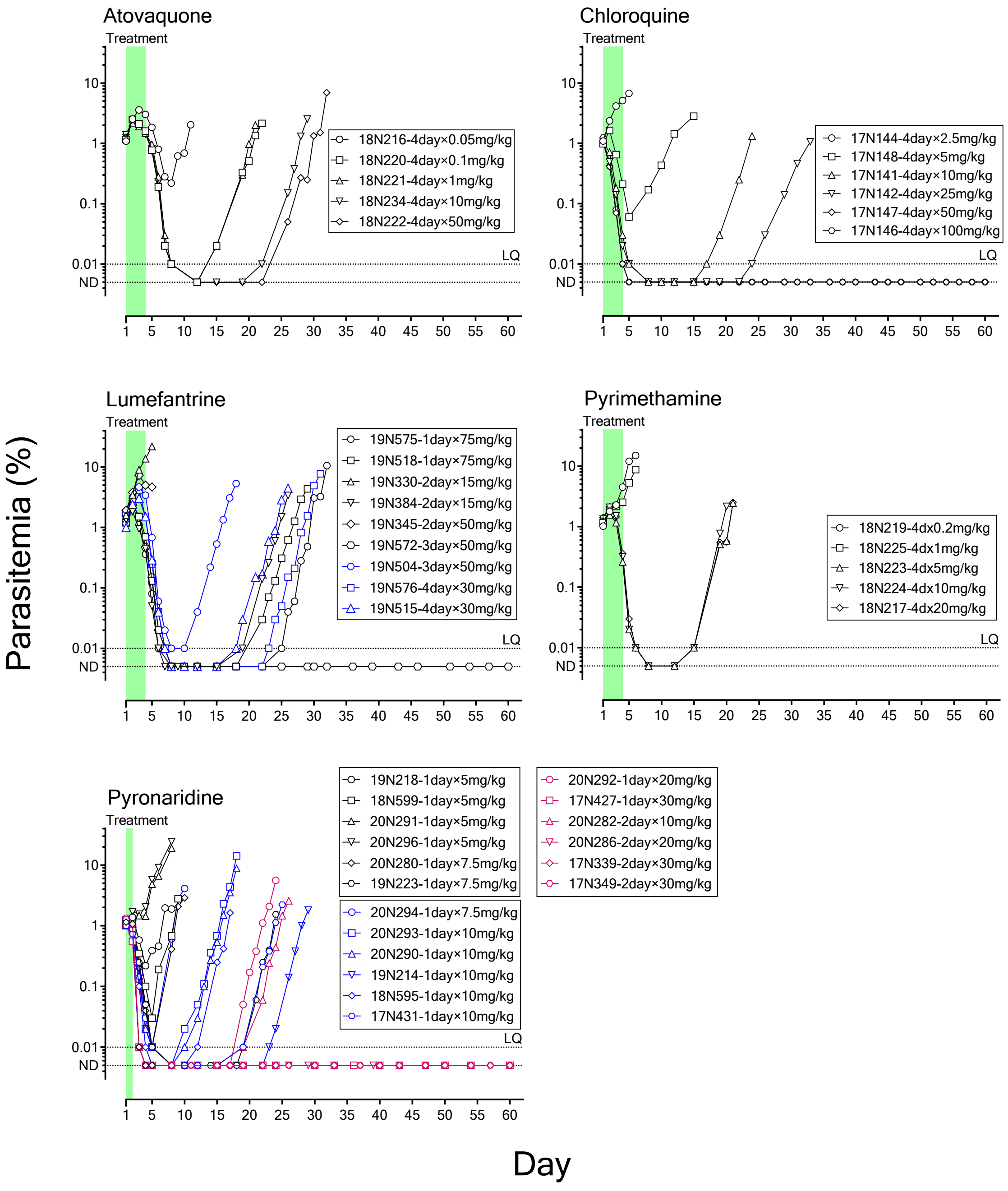

Supplementary Figure 1.
Parasitemia in peripheral blood of standard antimalarials. (a) The figure displays individual parasitemias over time, expressed as the % of *P. falciparum*-infected human erythrocytes with respect to the total (murine and human) circulating erythrocytes. ND indicates points at which parasitemia was below the limit of quantification (LQ) by flow cytometry. The green regions indicate the maximum number of days of drug administration.

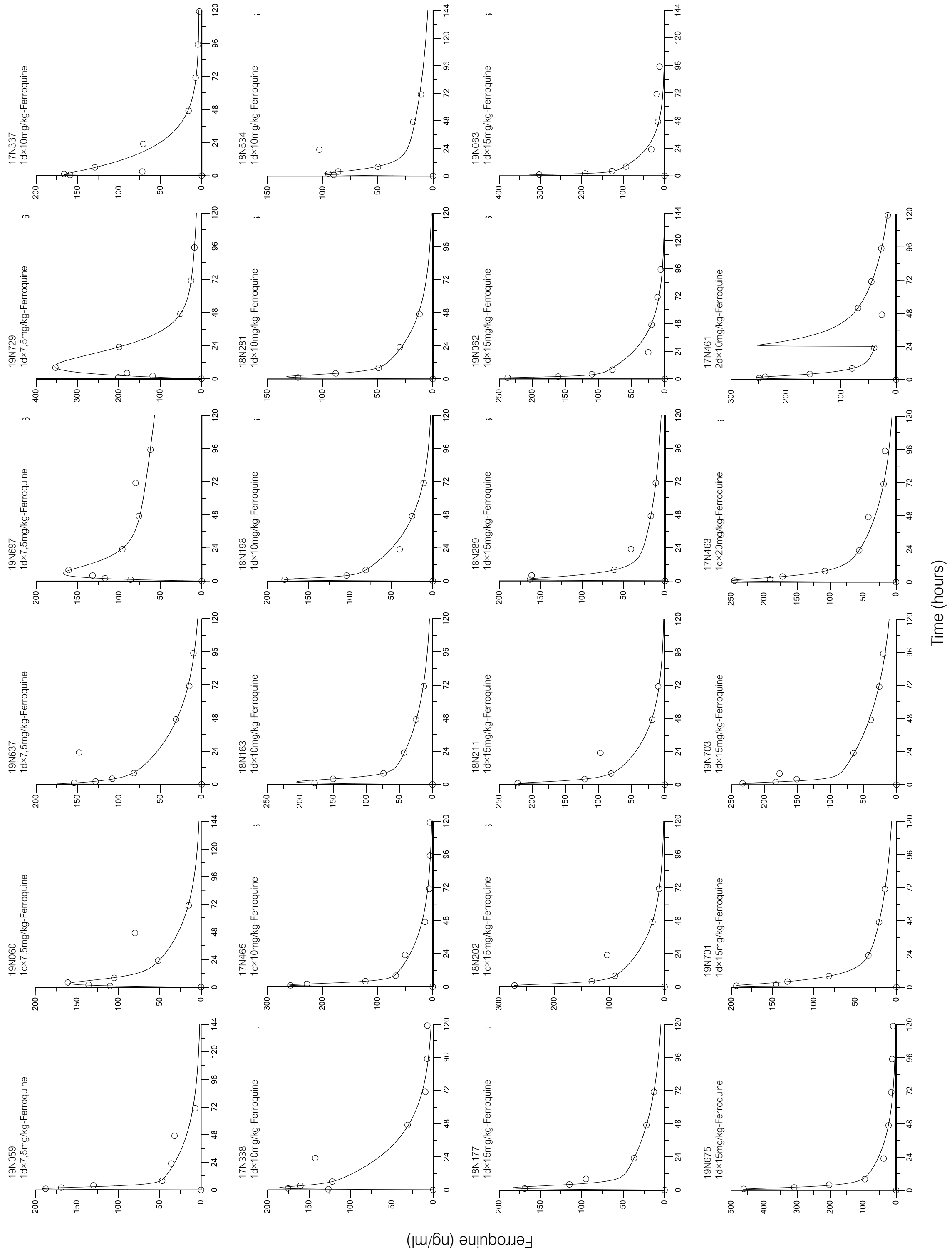

Supplementary Figure 2.

Concentration of ferroquine in peripheral blood of PfalcHuMice treated with ferroquine monotherapy. (a) The figure displays the concentration of ferroquine in ng/ml (open symbols). The lines are the fitted PK model for each individual. Each panel displays the unique identifier for individuals and the treatment that received.

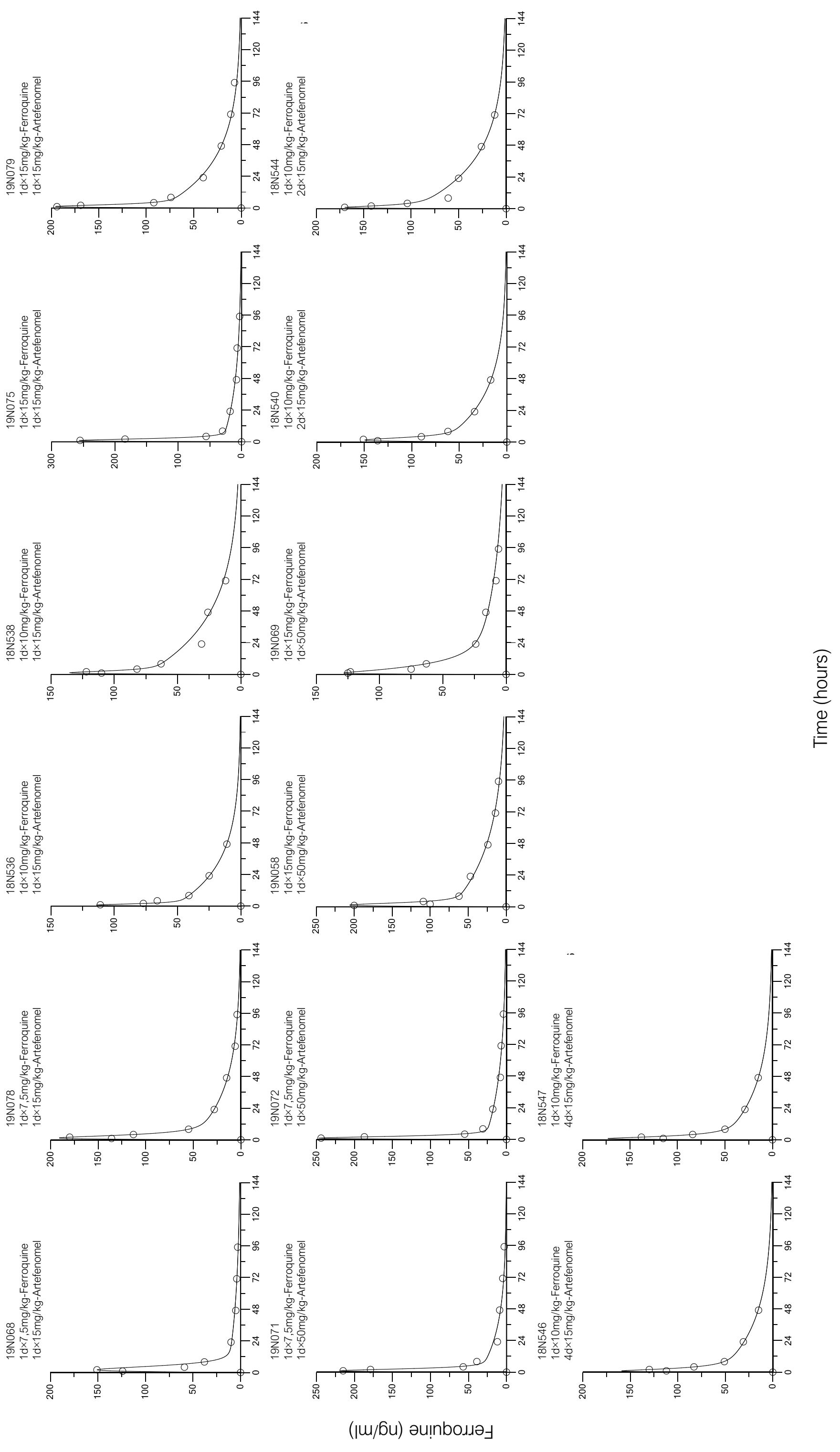

Supplementary Figure 3.

Concentration of ferroquine in peripheral blood of PfalcHuMice treated with ferroquine plus artefenomel. (a) The figure displays the concentration of ferroquine in ng/ml (open symbols). The lines are the fitted PK model for each individual. Each panel displays the unique identifier for individuals and the treatment that received.

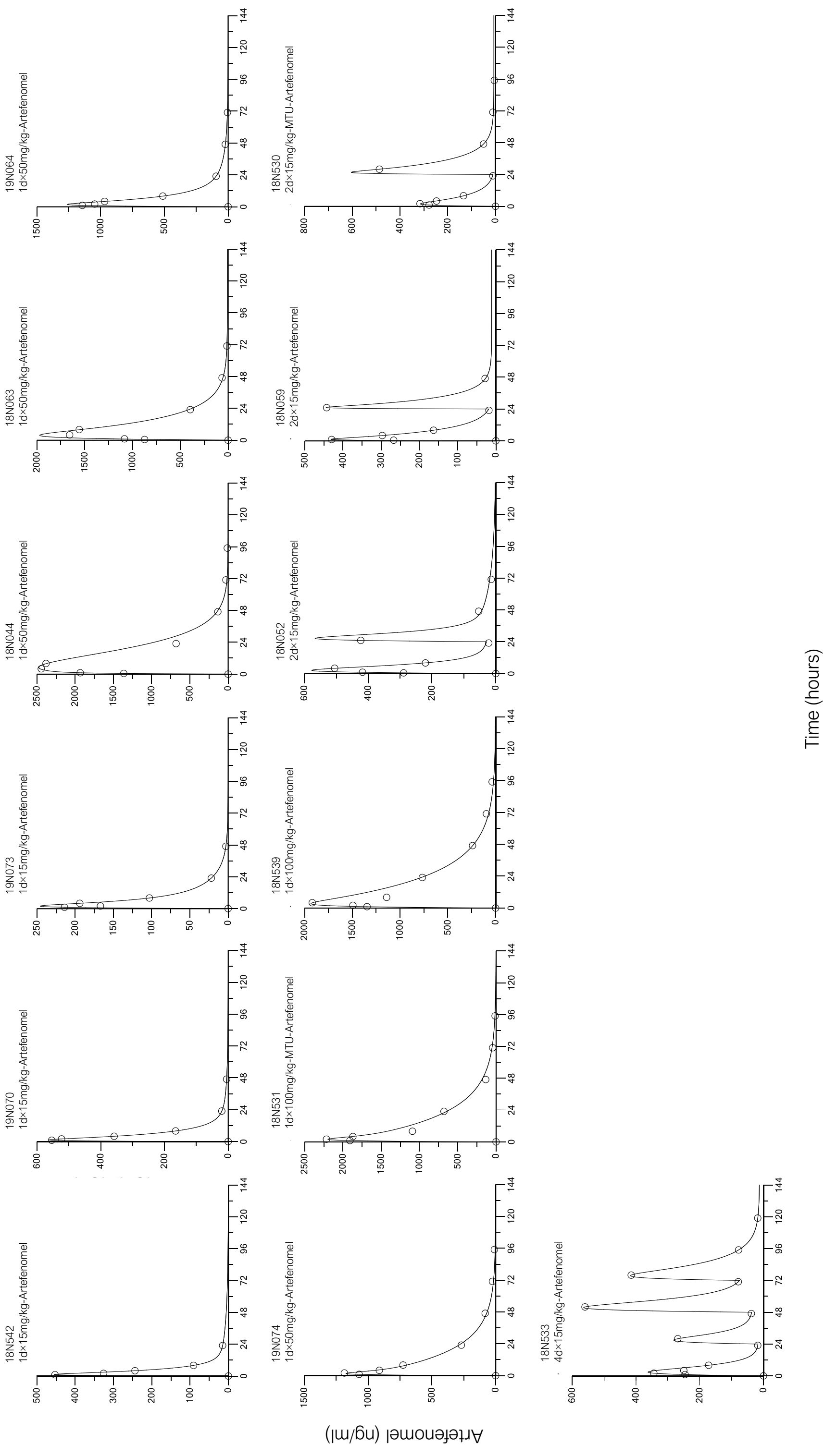

Supplementary Figure 4.

Concentration of artefenomel in peripheral blood of PfalcHuMice treated with artefenomel monotherapy. (a) The figure displays the concentration of artefenomel in ng/ml (open symbols). The lines are the fitted PK model for each individual. Each panel displays the unique identifier for individuals and the treatment that received.

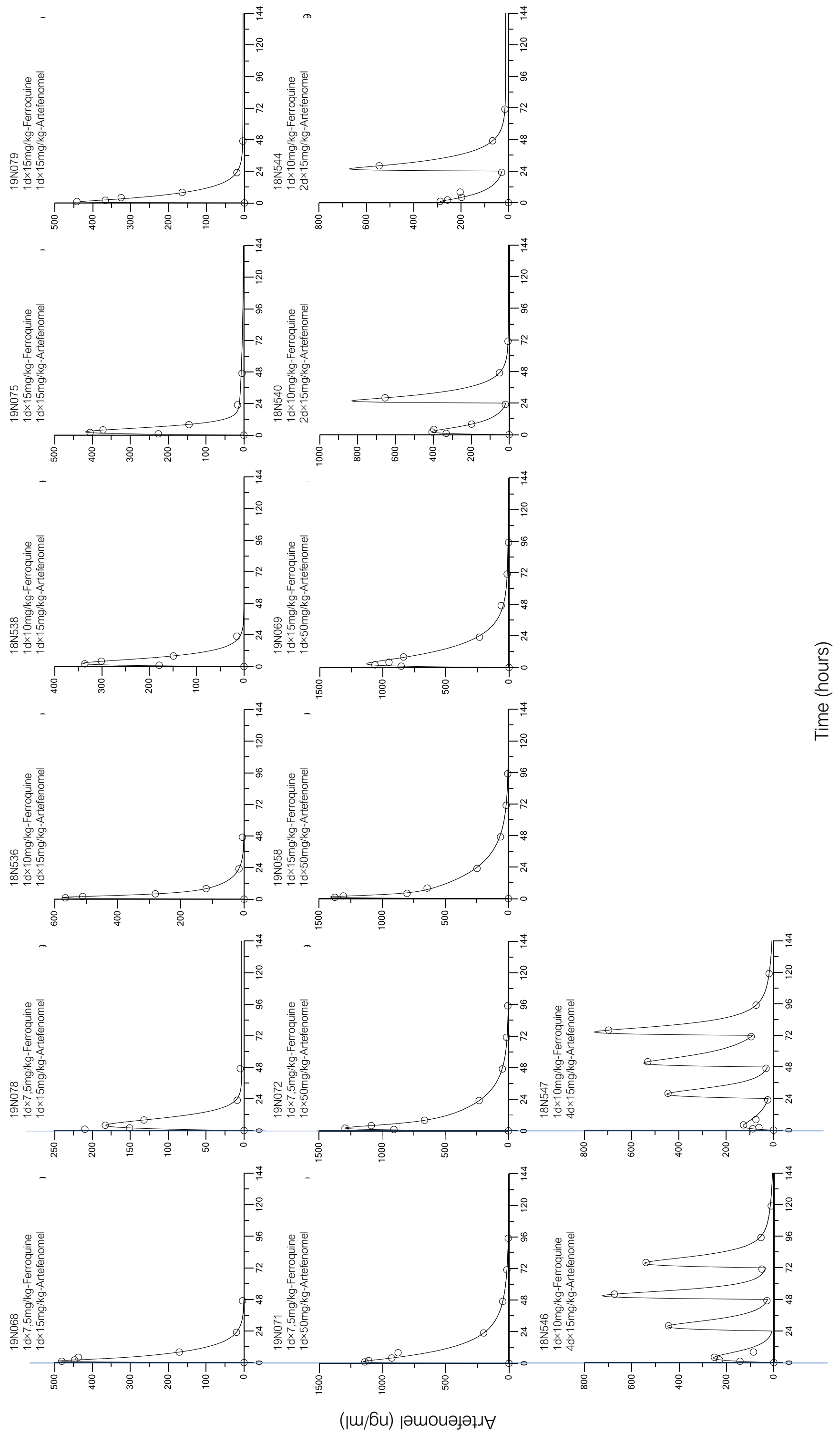

Supplementary Figure 5.

Concentration of artefenomel in peripheral blood of PfalcHuMice treated with ferroquine plus artefenomel. (a) The figure displays the concentration of artefenomel in ng/ml (open symbols). The lines are the fitted PK model for each individual. Each panel displays the unique identifier for individuals and the treatment that received.

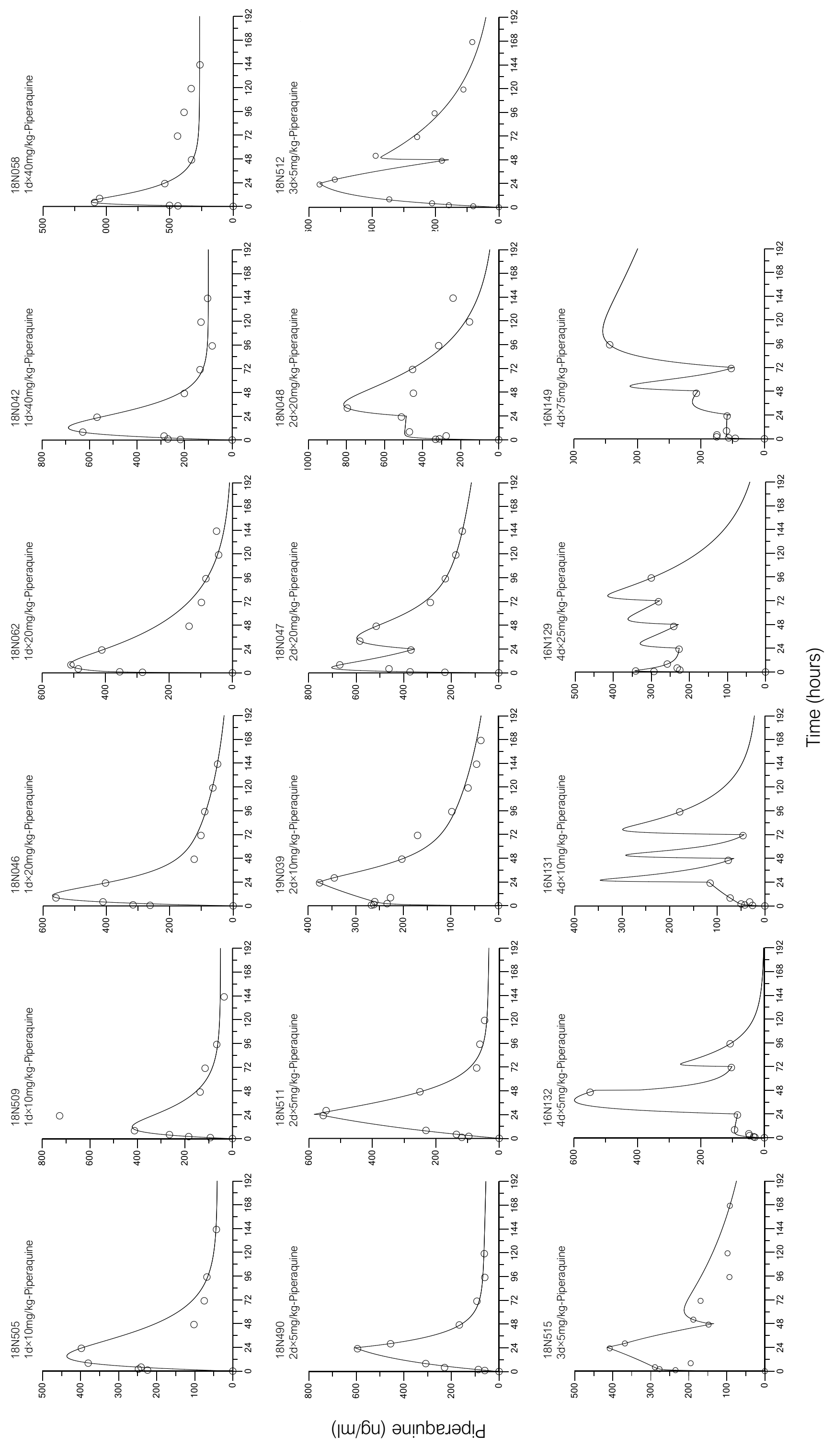

Supplementary Figure 6.

Concentration of piperaquine in peripheral blood of PfalcHuMice treated with piperaquine monotherapy. (a) The figure displays the concentration of piperaquine in ng/ml (open symbols). The lines are the fitted PK model for each individual. Each panel displays the unique identifier for individuals and the treatment that received.

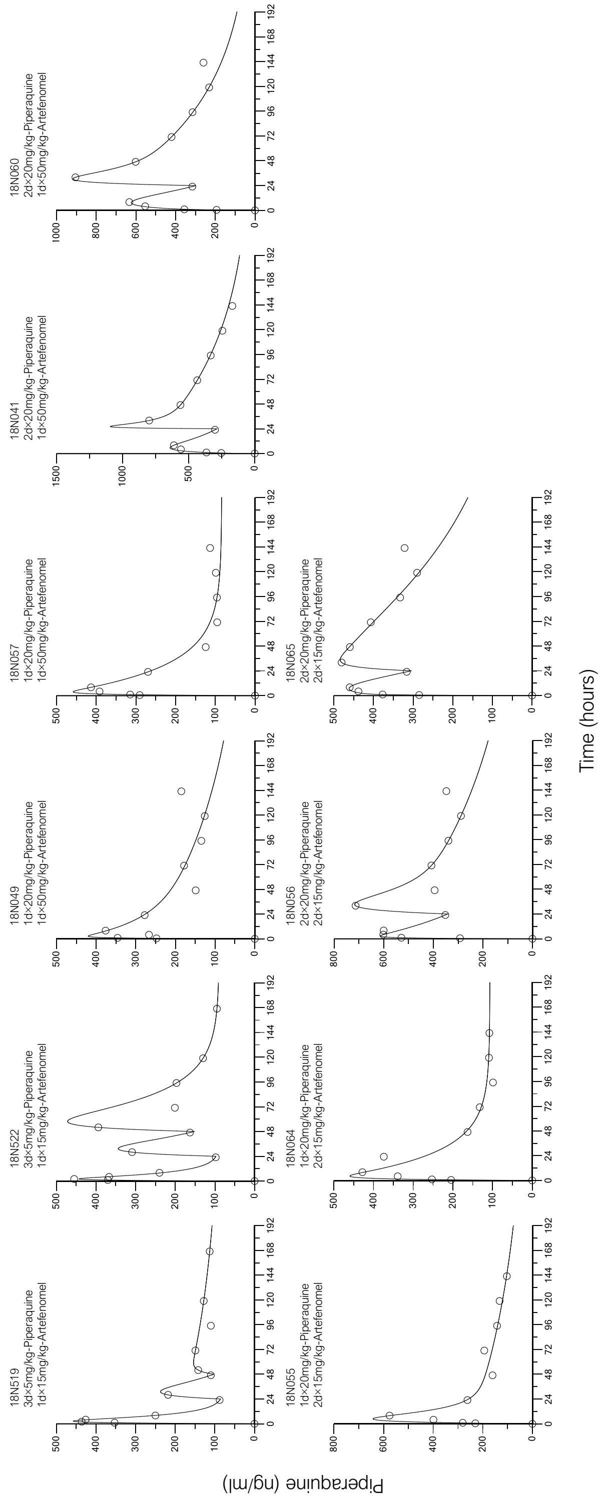

Supplementary Figure 7.

Concentration of piperaquine in peripheral blood of PfalcHuMice treated with piperaquine plus artefenomel. (a) The figure displays the concentration of artefenomel in ng/ml (open symbols). The lines are the fitted PK model for each individual. Each panel displays the unique identifier for individuals and the treatment that received.

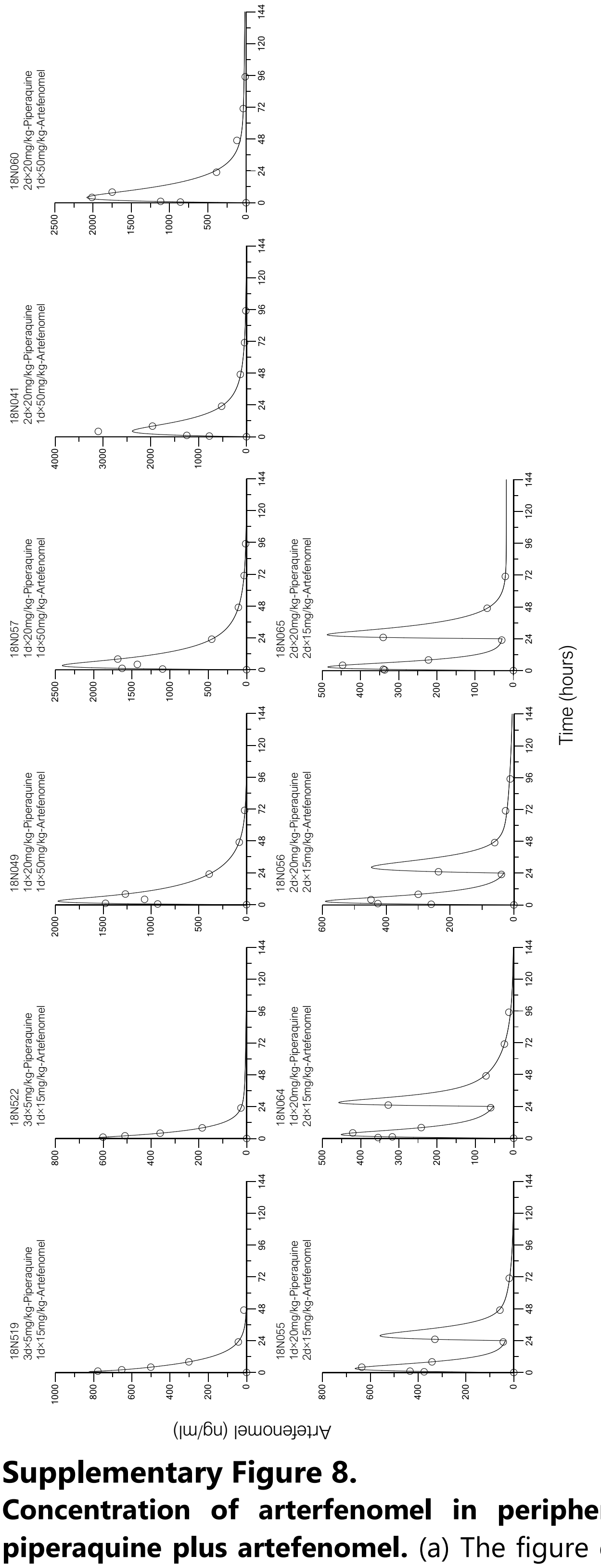

Supplementary Figure 8.

Concentration of arterfenomel in peripheral blood of PfalcHuMice treated with piperaquine plus artefenomel. (a) The figure displays the concentration of artefenomel in ng/ml (open symbols). The lines are the fitted PK model for each individual. Each panel displays the unique identifier for individuals and the treatment that received.

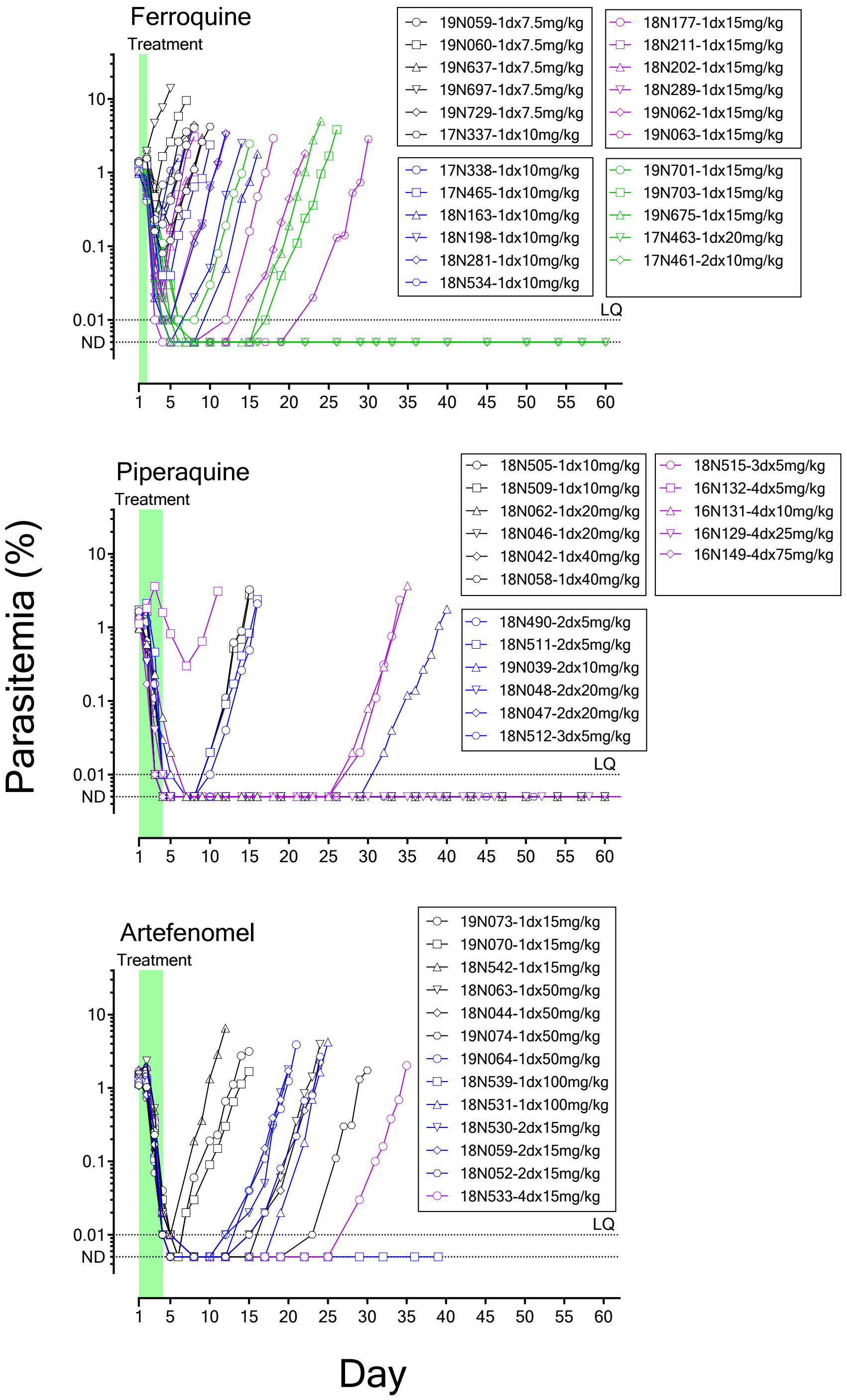

Supplementary Figure 9.
Parasitemia in peripheral blood of ferroquine, piperaquine and artefenomel. (a) The figure displays individual parasitemias over time, expressed as the % of *P. falciparum*-infected human erythrocytes with respect to the total (murine and human) circulating erythrocytes. ND indicates points at which parasitemia was below the limit of quantification (LQ) by flow cytometry. The green regions indicate the maximum number of days of drug administration.

MouseUniqueIdentifier	Analyte	Value	PKExplanatoryParameter	Unit
18N041	Artefenomel	4	T_{max}	hours
18N041	Artefenomel	2387	C_{max}	ng/ml
18N041	Artefenomel	41036	$AUC_{0toTlast}$	h×ng/ml
18N044	Artefenomel	5	T_{max}	hours
18N044	Artefenomel	2476	C_{max}	ng/ml
18N044	Artefenomel	56341	$AUC_{0toTlast}$	h×ng/ml
18N049	Artefenomel	3	T_{max}	hours
18N049	Artefenomel	1973	C_{max}	ng/ml
18N049	Artefenomel	29306	$AUC_{0toTlast}$	h×ng/ml
18N052	Artefenomel	3	T_{max}	hours
18N052	Artefenomel	577	C_{max}	ng/ml
18N052	Artefenomel	10599	$AUC_{0toTlast}$	h×ng/ml
18N055	Artefenomel	3	T_{max}	hours
18N055	Artefenomel	664	C_{max}	ng/ml
18N055	Artefenomel	13287	$AUC_{0toTlast}$	h×ng/ml
18N056	Artefenomel	3	T_{max}	hours
18N056	Artefenomel	591	C_{max}	ng/ml
18N056	Artefenomel	12718	$AUC_{0toTlast}$	h×ng/ml
18N057	Artefenomel	3	T_{max}	hours
18N057	Artefenomel	2410	C_{max}	ng/ml
18N057	Artefenomel	37154	$AUC_{0toTlast}$	h×ng/ml
18N059	Artefenomel	25	T_{max}	hours
18N059	Artefenomel	444	C_{max}	ng/ml
18N059	Artefenomel	8643	$AUC_{0toTlast}$	h×ng/ml
18N060	Artefenomel	4	T_{max}	hours
18N060	Artefenomel	2080	C_{max}	ng/ml
18N060	Artefenomel	36098	$AUC_{0toTlast}$	h×ng/ml
18N063	Artefenomel	4	T_{max}	hours
18N063	Artefenomel	1970	C_{max}	ng/ml
18N063	Artefenomel	31829	$AUC_{0toTlast}$	h×ng/ml
18N064	Artefenomel	27	T_{max}	hours
18N064	Artefenomel	457	C_{max}	ng/ml
18N064	Artefenomel	11171	$AUC_{0toTlast}$	h×ng/ml

18N065	Artefenomel	27	T_{max}	hours
18N065	Artefenomel	487	C_{max}	ng/ml
18N065	Artefenomel	12866	$AUC_{0toTlast}$	h×ng/ml
18N519	Artefenomel	1	T_{max}	hours
18N519	Artefenomel	824	C_{max}	ng/ml
18N519	Artefenomel	6481	$AUC_{0toTlast}$	h×ng/ml
18N522	Artefenomel	1	T_{max}	hours
18N522	Artefenomel	618	C_{max}	ng/ml
18N522	Artefenomel	4670	$AUC_{0toTlast}$	h×ng/ml
18N530	Artefenomel	26	T_{max}	hours
18N530	Artefenomel	604	C_{max}	ng/ml
18N530	Artefenomel	9565	$AUC_{0toTlast}$	h×ng/ml
18N531	Artefenomel	2	T_{max}	hours
18N531	Artefenomel	2196	C_{max}	ng/ml
18N531	Artefenomel	40914	$AUC_{0toTlast}$	h×ng/ml
18N533	Artefenomel	52	T_{max}	hours
18N533	Artefenomel	564	C_{max}	ng/ml
18N533	Artefenomel	19648	$AUC_{0toTlast}$	h×ng/ml
18N536	Artefenomel	1	T_{max}	hours
18N536	Artefenomel	575	C_{max}	ng/ml
18N536	Artefenomel	3323	$AUC_{0toTlast}$	h×ng/ml
18N538	Artefenomel	3	T_{max}	hours
18N538	Artefenomel	340	C_{max}	ng/ml
18N538	Artefenomel	2792	$AUC_{0toTlast}$	h×ng/ml
18N539	Artefenomel	3	T_{max}	hours
18N539	Artefenomel	1932	C_{max}	ng/ml
18N539	Artefenomel	46430	$AUC_{0toTlast}$	h×ng/ml
18N540	Artefenomel	26	T_{max}	hours
18N540	Artefenomel	831	C_{max}	ng/ml
18N540	Artefenomel	11868	$AUC_{0toTlast}$	h×ng/ml
18N542	Artefenomel	1	T_{max}	hours
18N542	Artefenomel	455	C_{max}	ng/ml
18N542	Artefenomel	2901	$AUC_{0toTlast}$	h×ng/ml
18N544	Artefenomel	26	T_{max}	hours
18N544	Artefenomel	671	C_{max}	ng/ml
18N544	Artefenomel	11294	$AUC_{0toTlast}$	h×ng/ml
18N546	Artefenomel	51	T_{max}	hours
18N546	Artefenomel	724	C_{max}	ng/ml
18N546	Artefenomel	20167	$AUC_{0toTlast}$	h×ng/ml
18N547	Artefenomel	75	T_{max}	hours

18N547	Artefenomel	757	C_{max}	ng/ml
18N547	Artefenomel	21762	$AUC_{0toTlast}$	h×ng/ml
19N058	Artefenomel	1	T_{max}	hours
19N058	Artefenomel	1410	C_{max}	ng/ml
19N058	Artefenomel	18017	$AUC_{0toTlast}$	h×ng/ml
19N064	Artefenomel	2	T_{max}	hours
19N064	Artefenomel	1262	C_{max}	ng/ml
19N064	Artefenomel	11958	$AUC_{0toTlast}$	h×ng/ml
19N068	Artefenomel	1	T_{max}	hours
19N068	Artefenomel	488	C_{max}	ng/ml
19N068	Artefenomel	3681	$AUC_{0toTlast}$	h×ng/ml
19N069	Artefenomel	3	T_{max}	hours
19N069	Artefenomel	1127	C_{max}	ng/ml
19N069	Artefenomel	19055	$AUC_{0toTlast}$	h×ng/ml
19N070	Artefenomel	1	T_{max}	hours
19N070	Artefenomel	563	C_{max}	ng/ml
19N070	Artefenomel	4068	$AUC_{0toTlast}$	h×ng/ml
19N071	Artefenomel	1	T_{max}	hours
19N071	Artefenomel	1154	C_{max}	ng/ml
19N071	Artefenomel	16224	$AUC_{0toTlast}$	h×ng/ml
19N072	Artefenomel	2	T_{max}	hours
19N072	Artefenomel	1294	C_{max}	ng/ml
19N072	Artefenomel	17272	$AUC_{0toTlast}$	h×ng/ml
19N073	Artefenomel	2	T_{max}	hours
19N073	Artefenomel	246	C_{max}	ng/ml
19N073	Artefenomel	2385	$AUC_{0toTlast}$	h×ng/ml
19N074	Artefenomel	2	T_{max}	hours
19N074	Artefenomel	1174	C_{max}	ng/ml
19N074	Artefenomel	19018	$AUC_{0toTlast}$	h×ng/ml
19N075	Artefenomel	3	T_{max}	hours
19N075	Artefenomel	418	C_{max}	ng/ml
19N075	Artefenomel	3563	$AUC_{0toTlast}$	h×ng/ml
19N078	Artefenomel	4	T_{max}	hours
19N078	Artefenomel	182	C_{max}	ng/ml
19N078	Artefenomel	2525	$AUC_{0toTlast}$	h×ng/ml
19N079	Artefenomel	1	T_{max}	hours
19N079	Artefenomel	432	C_{max}	ng/ml
19N079	Artefenomel	4042	$AUC_{0toTlast}$	h×ng/ml
17N337	Ferroquine	1	T_{max}	hours
17N337	Ferroquine	167	C_{max}	ng/ml

17N337	Ferroquine	3384	$AUC_{0toTlast}$	h×ng/ml
17N338	Ferroquine	2	T_{max}	hours
17N338	Ferroquine	186	C_{max}	ng/ml
17N338	Ferroquine	4268	$AUC_{0toTlast}$	h×ng/ml
17N461	Ferroquine	2	T_{max}	hours
17N461	Ferroquine	253	C_{max}	ng/ml
17N461	Ferroquine	8549	$AUC_{0toTlast}$	h×ng/ml
17N463	Ferroquine	2	T_{max}	hours
17N463	Ferroquine	246	C_{max}	ng/ml
17N463	Ferroquine	4708	$AUC_{0toTlast}$	h×ng/ml
17N465	Ferroquine	1	T_{max}	hours
17N465	Ferroquine	258	C_{max}	ng/ml
17N465	Ferroquine	3073	$AUC_{0toTlast}$	h×ng/ml
18N163	Ferroquine	2	T_{max}	hours
18N163	Ferroquine	205	C_{max}	ng/ml
18N163	Ferroquine	3490	$AUC_{0toTlast}$	h×ng/ml
18N177	Ferroquine	2	T_{max}	hours
18N177	Ferroquine	183	C_{max}	ng/ml
18N177	Ferroquine	3032	$AUC_{0toTlast}$	h×ng/ml
18N198	Ferroquine	1	T_{max}	hours
18N198	Ferroquine	179	C_{max}	ng/ml
18N198	Ferroquine	3371	$AUC_{0toTlast}$	h×ng/ml
18N202	Ferroquine	1	T_{max}	hours
18N202	Ferroquine	272	C_{max}	ng/ml
18N202	Ferroquine	3553	$AUC_{0toTlast}$	h×ng/ml
18N211	Ferroquine	1	T_{max}	hours
18N211	Ferroquine	222	C_{max}	ng/ml
18N211	Ferroquine	3124	$AUC_{0toTlast}$	h×ng/ml
18N281	Ferroquine	2	T_{max}	hours
18N281	Ferroquine	133	C_{max}	ng/ml
18N281	Ferroquine	1966	$AUC_{0toTlast}$	h×ng/ml
18N289	Ferroquine	2	T_{max}	hours
18N289	Ferroquine	164	C_{max}	ng/ml
18N289	Ferroquine	2588	$AUC_{0toTlast}$	h×ng/ml
18N534	Ferroquine	2	T_{max}	hours
18N534	Ferroquine	99	C_{max}	ng/ml
18N534	Ferroquine	2460	$AUC_{0toTlast}$	h×ng/ml
18N536	Ferroquine	1	T_{max}	hours
18N536	Ferroquine	111	C_{max}	ng/ml
18N536	Ferroquine	1548	$AUC_{0toTlast}$	h×ng/ml

18N538	Ferroquine	1	T_{max}	hours
18N538	Ferroquine	133	C_{max}	ng/ml
18N538	Ferroquine	3087	$AUC_{0toTlast}$	h×ng/ml
18N540	Ferroquine	2	T_{max}	hours
18N540	Ferroquine	150	C_{max}	ng/ml
18N540	Ferroquine	2411	$AUC_{0toTlast}$	h×ng/ml
18N544	Ferroquine	1	T_{max}	hours
18N544	Ferroquine	170	C_{max}	ng/ml
18N544	Ferroquine	3383	$AUC_{0toTlast}$	h×ng/ml
18N546	Ferroquine	1	T_{max}	hours
18N546	Ferroquine	159	C_{max}	ng/ml
18N546	Ferroquine	2195	$AUC_{0toTlast}$	h×ng/ml
18N547	Ferroquine	1	T_{max}	hours
18N547	Ferroquine	173	C_{max}	ng/ml
18N547	Ferroquine	2162	$AUC_{0toTlast}$	h×ng/ml
19N058	Ferroquine	2	T_{max}	hours
19N058	Ferroquine	204	C_{max}	ng/ml
19N058	Ferroquine	3471	$AUC_{0toTlast}$	h×ng/ml
19N059	Ferroquine	1	T_{max}	hours
19N059	Ferroquine	188	C_{max}	ng/ml
19N059	Ferroquine	2491	$AUC_{0toTlast}$	h×ng/ml
19N060	Ferroquine	3	T_{max}	hours
19N060	Ferroquine	158	C_{max}	ng/ml
19N060	Ferroquine	3918	$AUC_{0toTlast}$	h×ng/ml
19N062	Ferroquine	1	T_{max}	hours
19N062	Ferroquine	236	C_{max}	ng/ml
19N062	Ferroquine	3199	$AUC_{0toTlast}$	h×ng/ml
19N063	Ferroquine	1	T_{max}	hours
19N063	Ferroquine	321	C_{max}	ng/ml
19N063	Ferroquine	3053	$AUC_{0toTlast}$	h×ng/ml
19N068	Ferroquine	2	T_{max}	hours
19N068	Ferroquine	151	C_{max}	ng/ml
19N068	Ferroquine	1224	$AUC_{0toTlast}$	h×ng/ml
19N069	Ferroquine	1	T_{max}	hours
19N069	Ferroquine	128	C_{max}	ng/ml
19N069	Ferroquine	2374	$AUC_{0toTlast}$	h×ng/ml
19N071	Ferroquine	1	T_{max}	hours
19N071	Ferroquine	219	C_{max}	ng/ml
19N071	Ferroquine	1449	$AUC_{0toTlast}$	h×ng/ml
19N072	Ferroquine	1	T_{max}	hours

19N072	Ferroquine	247	C_{max}	ng/ml
19N072	Ferroquine	1639	$AUC_{0toTlast}$	h×ng/ml
19N075	Ferroquine	1	T_{max}	hours
19N075	Ferroquine	256	C_{max}	ng/ml
19N075	Ferroquine	1609	$AUC_{0toTlast}$	h×ng/ml
19N078	Ferroquine	2	T_{max}	hours
19N078	Ferroquine	191	C_{max}	ng/ml
19N078	Ferroquine	2267	$AUC_{0toTlast}$	h×ng/ml
19N079	Ferroquine	1	T_{max}	hours
19N079	Ferroquine	195	C_{max}	ng/ml
19N079	Ferroquine	2970	$AUC_{0toTlast}$	h×ng/ml
19N637	Ferroquine	1	T_{max}	hours
19N637	Ferroquine	167	C_{max}	ng/ml
19N637	Ferroquine	3871	$AUC_{0toTlast}$	h×ng/ml
19N675	Ferroquine	1	T_{max}	hours
19N675	Ferroquine	453	C_{max}	ng/ml
19N675	Ferroquine	4273	$AUC_{0toTlast}$	h×ng/ml
19N697	Ferroquine	6	T_{max}	hours
19N697	Ferroquine	167	C_{max}	ng/ml
19N697	Ferroquine	24563	$AUC_{0toTlast}$	h×ng/ml
19N701	Ferroquine	1	T_{max}	hours
19N701	Ferroquine	194	C_{max}	ng/ml
19N701	Ferroquine	3334	$AUC_{0toTlast}$	h×ng/ml
19N703	Ferroquine	1	T_{max}	hours
19N703	Ferroquine	233	C_{max}	ng/ml
19N703	Ferroquine	5400	$AUC_{0toTlast}$	h×ng/ml
19N729	Ferroquine	9	T_{max}	hours
19N729	Ferroquine	355	C_{max}	ng/ml
19N729	Ferroquine	11081	$AUC_{0toTlast}$	h×ng/ml
16N129	Piperaquine	77	T_{max}	hours
16N129	Piperaquine	414	C_{max}	ng/ml
16N129	Piperaquine	41945	$AUC_{0toTlast}$	h×ng/ml
16N131	Piperaquine	26	T_{max}	hours
16N131	Piperaquine	346	C_{max}	ng/ml
16N131	Piperaquine	21817	$AUC_{0toTlast}$	h×ng/ml
16N132	Piperaquine	38	T_{max}	hours
16N132	Piperaquine	598	C_{max}	ng/ml
16N132	Piperaquine	25304	$AUC_{0toTlast}$	h×ng/ml
16N149	Piperaquine	109	T_{max}	hours
16N149	Piperaquine	2546	C_{max}	ng/ml

16N149	Piperaquine	354505	$AUC_{0toTlast}$	h×ng/ml
18N041	Piperaquine	26	T_{max}	hours
18N041	Piperaquine	1092	C_{max}	ng/ml
18N041	Piperaquine	70376	$AUC_{0toTlast}$	h×ng/ml
18N042	Piperaquine	13	T_{max}	hours
18N042	Piperaquine	689	C_{max}	ng/ml
18N042	Piperaquine	38866	$AUC_{0toTlast}$	h×ng/ml
18N046	Piperaquine	11	T_{max}	hours
18N046	Piperaquine	570	C_{max}	ng/ml
18N046	Piperaquine	28190	$AUC_{0toTlast}$	h×ng/ml
18N047	Piperaquine	6	T_{max}	hours
18N047	Piperaquine	703	C_{max}	ng/ml
18N047	Piperaquine	57634	$AUC_{0toTlast}$	h×ng/ml
18N048	Piperaquine	36	T_{max}	hours
18N048	Piperaquine	814	C_{max}	ng/ml
18N048	Piperaquine	64302	$AUC_{0toTlast}$	h×ng/ml
18N049	Piperaquine	3	T_{max}	hours
18N049	Piperaquine	420	C_{max}	ng/ml
18N049	Piperaquine	33457	$AUC_{0toTlast}$	h×ng/ml
18N055	Piperaquine	5	T_{max}	hours
18N055	Piperaquine	642	C_{max}	ng/ml
18N055	Piperaquine	33863	$AUC_{0toTlast}$	h×ng/ml
18N056	Piperaquine	34	T_{max}	hours
18N056	Piperaquine	717	C_{max}	ng/ml
18N056	Piperaquine	71935	$AUC_{0toTlast}$	h×ng/ml
18N057	Piperaquine	4	T_{max}	hours
18N057	Piperaquine	458	C_{max}	ng/ml
18N057	Piperaquine	27943	$AUC_{0toTlast}$	h×ng/ml
18N058	Piperaquine	5	T_{max}	hours
18N058	Piperaquine	1120	C_{max}	ng/ml
18N058	Piperaquine	69984	$AUC_{0toTlast}$	h×ng/ml
18N060	Piperaquine	31	T_{max}	hours
18N060	Piperaquine	917	C_{max}	ng/ml
18N060	Piperaquine	67933	$AUC_{0toTlast}$	h×ng/ml
18N062	Piperaquine	9	T_{max}	hours
18N062	Piperaquine	512	C_{max}	ng/ml
18N062	Piperaquine	28569	$AUC_{0toTlast}$	h×ng/ml
18N064	Piperaquine	5	T_{max}	hours
18N064	Piperaquine	459	C_{max}	ng/ml
18N064	Piperaquine	31139	$AUC_{0toTlast}$	h×ng/ml

18N065	Piperaquine	35	T_{max}	hours
18N065	Piperaquine	484	C_{max}	ng/ml
18N065	Piperaquine	64153	$AUC_{0toTlast}$	h×ng/ml
18N490	Piperaquine	24	T_{max}	hours
18N490	Piperaquine	606	C_{max}	ng/ml
18N490	Piperaquine	27321	$AUC_{0toTlast}$	h×ng/ml
18N505	Piperaquine	15	T_{max}	hours
18N505	Piperaquine	436	C_{max}	ng/ml
18N505	Piperaquine	25838	$AUC_{0toTlast}$	h×ng/ml
18N509	Piperaquine	12	T_{max}	hours
18N509	Piperaquine	421	C_{max}	ng/ml
18N509	Piperaquine	23624	$AUC_{0toTlast}$	h×ng/ml
18N511	Piperaquine	25	T_{max}	hours
18N511	Piperaquine	580	C_{max}	ng/ml
18N511	Piperaquine	26393	$AUC_{0toTlast}$	h×ng/ml
18N512	Piperaquine	24	T_{max}	hours
18N512	Piperaquine	566	C_{max}	ng/ml
18N512	Piperaquine	41254	$AUC_{0toTlast}$	h×ng/ml
18N515	Piperaquine	24	T_{max}	hours
18N515	Piperaquine	415	C_{max}	ng/ml
18N515	Piperaquine	35575	$AUC_{0toTlast}$	h×ng/ml
18N519	Piperaquine	3	T_{max}	hours
18N519	Piperaquine	457	C_{max}	ng/ml
18N519	Piperaquine	28652	$AUC_{0toTlast}$	h×ng/ml
18N522	Piperaquine	58	T_{max}	hours
18N522	Piperaquine	471	C_{max}	ng/ml
18N522	Piperaquine	39040	$AUC_{0toTlast}$	h×ng/ml
19N039	Piperaquine	26	T_{max}	hours
19N039	Piperaquine	370	C_{max}	ng/ml
19N039	Piperaquine	22337	$AUC_{0toTlast}$	h×ng/ml
T_{max}: Time of maximum concentration in samples from 0 to last hour of sampling				
C_{max}: Maximum concentration in biological samples taken from 0 to last time of sampling				
$AUC_{0toTlast}$: Area under the curve of the drug in individuals from treatment inception (t=0) to last time of sampling (T_{last})				

Supplementary Table 1.
Primary explanatory pharmacokinetic parameters in the individuals of the combination studies reported in this paper.
